# Gut Microbiota Metabolites and Chronic Diseases: Interactions, Mechanisms, and Therapeutic Strategies

**DOI:** 10.3390/ijms26083752

**Published:** 2025-04-16

**Authors:** Wenwen Liu, Lei Wang, Jinmei Ou, Daiyin Peng, Yue Zhang, Weidong Chen, Yanyan Wang

**Affiliations:** 1 School of Pharmacy, Anhui University of Chinese Medicine, Hefei 230012, China; liuwenwen117@163.com (W.L.); wanglei@ahtcm.edu.cn (L.W.); ojm@ahtcm.edu.cn (J.O.); pengdaiyin@163.com (D.P.); wdchen@ahtcm.edu.cn (W.C.); 2 Ministry of Education-Anhui Joint Collaborative Innovation Center for Quality Improvement of Anhui Genuine Chinese Medicinal Materials, Hefei 230012, China; 3 Institute of Traditional Chinese Medicine Resources Protection and Development, Hefei 230012, China; 4 Anhui Key Laboratory of New Manufacturing Technology of Chinese Medicine Pieces, Hefei 230012, China

**Keywords:** gut microbiota, metabolites, chronic diseases

## Abstract

The gut microbiota, shaped by factors such as diet, lifestyle, and genetics, plays a pivotal role in regulating host metabolism, immune function, and overall health. The diversity and balance of the gut microbiota are closely linked to the onset and progression of various chronic diseases. A growing body of evidence has demonstrated that alterations in the composition, function, and metabolites of the gut microbiota are significantly associated with cardiovascular diseases, including hypertension, atherosclerosis, and heart failure; metabolic disorders such as obesity, type 2 diabetes, and metabolic dysfunction-associated steatotic liver disease; and gastrointestinal conditions like inflammatory bowel disease and colorectal cancer. Despite substantial advances in microbiome research, challenges remain in fully elucidating the causal relationships between the gut microbiota and disease, as well as in translating these insights into clinical applications. This review aims to investigate the regulatory pathways via which the gut microbiota affects cardiovascular health, metabolic function, and gastrointestinal disease. Additionally, it highlights emerging strategies for the prevention and treatment of these chronic conditions, focusing on microbiota-targeted therapies and personalized dietary interventions as promising approaches for improving health outcomes.

## 1. Introduction

Chronic diseases, also referred to as non-communicable diseases (NCDs), are characterized by insidious onset, prolonged progression, and complex etiologies. These include cardiovascular diseases (CVDs), metabolic disorders, and gastrointestinal conditions, which are often strongly influenced by lifestyle and environmental factors [1]. According to statistics from the World Health Organization, NCDs are responsible for approximately 41 million deaths annually, accounting for 74% of global mortality. This burden is particularly severe in low- and middle-income countries, where over three-quarters of NCD-related deaths occur.

**Figure 1:**
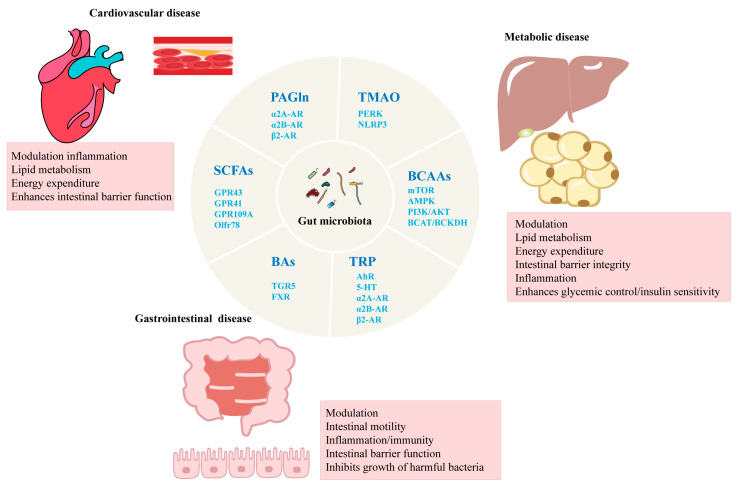
Schematic diagram of role of gut microbiota and its metabolites in regulating chronic diseases.

In recent years, advancements in molecular biology, genomics, and metabolomics have brought the gut microbiota into the spotlight in medical and biological research [2,3]. Although dysbiosis can accelerate disease progression by producing harmful metabolites and inducing inflammatory responses, emerging evidence suggests that a diverse gut microbiota is vital for human health. A healthy microbiota supports host metabolic function by enhancing intestinal barrier integrity, optimizing energy extraction, protecting against pathogens, and modulating the immune system to reduce systemic inflammation [4,5,6]. Key metabolites produced by the gut microbiota, such as trimethylamine N-oxide (TMAO), short-chain fatty acids (SCFAs), bile acids (BAs), phenylacetylglutamine (PAGln), branched-chain amino acids (BCAAs), and tryptophan (TRP), act as crucial “bridges” linking microbiota composition with disease, playing significant roles in the pathogenesis of microbiota-related diseases [7] (Figure 1). This study summarizes the link between gut microbiota metabolites and chronic diseases, as shown in Table 1.

With the rapid evolution of technologies such as amplicon sequencing, high-throughput sequencing, single-cell genomics, and CRISPR-Cas9 gene editing, innovative research methods targeting the gut microbiota are constantly emerging, offering avenues for developing diagnostic tools and therapeutic strategies. This review aims to summarize the roles and mechanisms by which the gut microbiota and its metabolites contribute to the pathogenesis of chronic diseases and discusses the potential of microbiota-targeted approaches in the prevention and treatment of these conditions.

## 2. Gut Microbiota and Metabolites

The description of microorganisms by humans dates back to the 17th century with Antonie van Leeuwenhoek, and the origins of the gut microbiota can be traced to the early 20th century. Scientists have studied the composition and function of the gut microbiota from the perspectives of microbiology and nutrition (Milestones in Human Microbiota Research—Nature https://www.nature.com/immersive/d42859-019-00041-z/index.html accessed on 25 November 2024). From the 1970s to the early 21st century, with the development of molecular biology, researchers began using 16S rRNA gene sequencing to classify and identify gut microbes, revealing that the gut microbiota is primarily composed of bacteria. The majority belong to the phyla *Firmicutes*, *Bacteroidetes*, *Actinobacteria*, and *Proteobacteria*. Within each phylum, multiple genera are found, and each genus contains various species, forming a highly complex microbial network [29,30].

In 1958, Eiseman’s report on the successful treatment of pseudomembranous colitis using fecal microbiota transplantation (FMT) with healthy human fecal material established the foundation for microbiome therapy [31]. In 1965, Schaedler and colleagues were the first to transplant bacteria into germ-free mice, revealing the impact of the gut microbiota on host development and physiological functions [32]. The homeostasis of the gut microbiota is crucial for maintaining human health. A diverse gut microbiota is generally associated with good health, while reduced diversity has been linked to a range of diseases, including inflammatory bowel disease (IBD), obesity, and metabolic disorders [33].

In an experimental study involving *Escherichia coli* infection in mice, the transplantation of normal mouse fecal microbiota into infected mice effectively reduced intestinal damage and mortality in the infected mice to some extent [34]. Similarly, in patients with type 1 diabetes, fecal microbiota transplantation from healthy donors into recently diagnosed patients halted the decline in endogenous insulin production [35]. Furthermore, studies have shown that changes in the gut microbiota are closely associated with the onset of obesity and insulin resistance. Germ-free mice transplanted with microbiota from obese mice exhibited significantly increased body weight and fat mass compared to those transplanted with normal microbiota, highlighting the potential of the gut microbiota in treating various diseases [36,37]. Extensive research demonstrates that the gut microbiota plays a crucial role in the development and progression of diseases by producing metabolites that influence gut barrier function and modulate the host immune response [38].

### 2.1. TMAO

TMAO is metabolized to trimethylamine (TMA) by cholinergics in red meat and dairy products by intestinal microorganisms (e.g., *Anaerococcus hydrogenalis*, *Clostridium asparagiforme*, *Clostridium Hathewayi,* etc.), and then by flavin monooxygenases (FMOs) in the host’s liver metabolites generated by oxidation. Elevated levels are significantly associated with cardiovascular disease risk by mechanisms involving the promotion of atherosclerotic plaque formation and platelet activation [39,40]. Epidemiological studies have shown that elevated serum levels of TMAO are closely associated with the development of metabolic diseases and other chronic conditions, including gastrointestinal disorders, suggesting that TMAO may serve as a potential biomarker for disease diagnosis and therapeutic intervention.

### 2.2. SCFAs

SCFAs (acetic acid/propionic acid/butyric acid) are produced by fermentation of dietary fiber by *Akkermansia muciniphila* and *Blautia hydrogenotrophica*, and regulate energy metabolism and immune homeostasis by activating receptors such as FFAR2/3. Butyric acid reduces inflammatory bowel disease by enhancing intestinal barrier function; propionic acid improves glucose metabolism through GLP-1 secretion, and reduced levels are associated with obesity and type 2 diabetes mellitus [41,42,43,44]. Recent studies suggest that SCFAs have potential in colorectal cancer control by influencing the tumor microenvironment through epigenetic regulation.

### 2.3. BAs

Gut flora converts primary bile acids to secondary bile acids via 7α dehydroxylation, and regulates lipid metabolism and inflammatory responses via FXR/TGR5 receptors. Imbalance in BAs composition correlates with the progression of NAFLD/NASH, whereas goose deoxycholic acid (UDCA), among others, improves the metabolic syndrome by inhibiting hepatic inflammation. Notably, BAs-TGR5 axis activation regulates myocardial energy metabolism, providing new ideas for heart failure treatment [45,46,47,48].

### 2.4. PAGln

Derived from the metabolism of phenylalanine by intestinal flora, PAGln directly promotes thrombosis and risk of myocardial infarction by enhancing platelet activity through activation of adrenergic receptors. The abundance of strains carrying the porA gene (e.g., *Clostridium* spp.) positively correlates with PAGln levels, and targeting this metabolic pathway may be a new strategy for antithrombotic therapy [49,50].

### 2.5. BCAAs

Elevated levels of circulating BCAAs are associated with insulin resistance and heart failure progression. The mechanism involves inhibition of AMPK signaling and mitochondrial dysfunction, while gut flora influence host metabolism by regulating BCAT/BCKD enzyme activity. Clinical studies have found that supplementation with probiotics to regulate the breakdown of BCAAs improves glucolipid metabolism in diabetic patients [51,52,53].

### 2.6. TRP

The intestinal flora regulates TRP metabolism through two pathways, kynurenine/indole: (i) IDO1-mediated activation of the kynurenine pathway is associated with depression and immune tolerance, and its metabolites promote atherosclerosis; and (ii) indole derivatives maintain the intestinal barrier and inhibit inflammation through the AHR, and are protective in IBD and autoimmune diseases. Abnormalities in the serotonin pathway, on the other hand, are closely associated with gut–brain axis dysfunction [54,55,56,57,58,59].

## 3. The Relationship Between Gut Microbiota and Chronic Diseases

### 3.1. CVD

CVDs are the leading cause of morbidity and mortality worldwide, primarily resulting from a combination of socioeconomic, metabolic, behavioral, and environmental risk factors [60]. The latest survey reports indicate that more than 500 million people worldwide are affected by CVDs, with approximately 17.9 million deaths each year, accounting for 32% of global mortality, and approximately 20.5 million deaths from CVDs in 2021 [60,61]. These diseases mainly include coronary artery disease, hypertension (HTN), and myocardial infarction, with atherosclerotic cardiovascular disease accounting for 80% of cardiovascular deaths [62]. CVDs not only impose a significant health burden but also come with heavy economic costs, with the annual economic burden of CVDs exceeding 200 billion euros in Europe [63,64].

Although lifestyle improvements and medical interventions can effectively prevent and control CVDs, the global mortality rate from CVDs continues to rise, especially in low- and middle-income countries. Therefore, early identification and management of CVD risks, as well as the development of novel therapeutic strategies, are critical for improving patient prognosis and reducing the global burden of CVDs.

**Figure 2:**
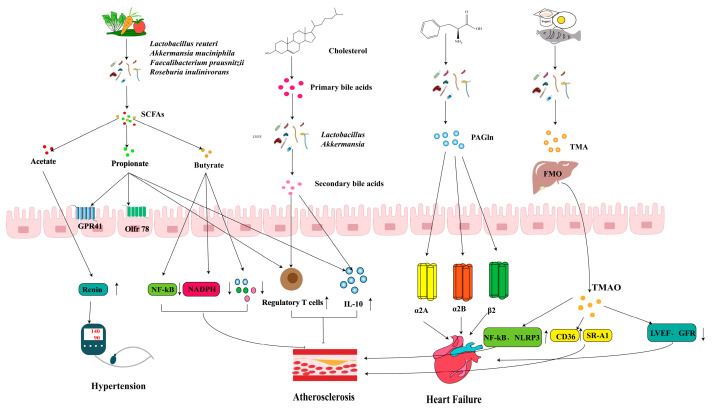
Mechanisms of action of gut microbes and their metabolites to ameliorate cardiovascular disease.

There is a complex interaction between the gut microbiota and CVDs. Dysbiosis of the gut microbiota has been implicated in various CVDs, including atherosclerosis (AS), HTN, and heart failure (HF). The gut microbiota metabolize specific dietary components to produce metabolites such as TMAO, PAGln, BAs, and SCFAs (Figure 2). The levels of these metabolites in the blood are associated with the risk of developing CVD and influence various physiological functions in the host, including blood pressure regulation, inflammatory responses, lipid metabolism, and endothelial function [65].

#### 3.1.1. HFN

HTN is one of the most common chronic NCDs and is the primary risk factor for CVD. It is increasingly affecting younger populations, and numerous studies have demonstrated that the gut microbiota and its metabolites are closely associated with blood pressure. Systematic reviews and meta-analyses have found that hypertensive patients exhibit significantly lower gut microbiota richness and diversity compared to healthy controls, with a notable increase in the *Firmicutes*/*Bacteroidetes* ratio, and at the genus level, compared to healthy controls, hypertensive patients show a significant reduction in the relative abundance of *Faecalibacterium* and an increase in the relative abundance of *Streptococcus* and *Enterococcus*, suggesting that HTN may be associated with gut dysbiosis [66].

By transplanting the fecal microbiota from hypertensive patients into germ-free mice, an increase in blood pressure was observed, confirming the direct effect of the gut microbiota on the host’s blood pressure [67]. Dysbiosis of the gut microbiota enhanced sympathetic nerve connections between the gut and the paraventricular nucleus (PVN) of the hypothalamus. When the gut microbiota from healthy rats was transplanted into hypertensive rats, inflammation and sympathetic stimulation in the PVN were reduced, leading to a decrease in blood pressure. This suggests that gut microbiota metabolism may influence sympathetic drive through gut-sympathetic nervous interactions or by promoting neuroinflammation, thereby affecting blood pressure [68].

Long-term exposure to cold conditions typically causes elevated blood pressure. Studies have shown that supplementing with sodium butyrate promotes the growth of beneficial bacteria such as *Lactobacillaceae* and reduces the abundance of harmful bacteria such as *Actinobacteriota* and *Erysipelotrichaceae*. It also activates Peroxisome proliferator-activated receptor γ coactivator lalpha and fibroblast growth factor21 levels in brown adipose tissue, lowering angiotensin II (ANG II) levels in the hypertensive group [69]. Furthermore, when sodium butyrate was administered to pregnant mothers fed a TRP-free diet, the results showed that sodium butyrate could prevent HTN in the offspring by increasing the expression of GPR41 and GPR109A in the kidneys and restoring the balance of the renin-angiotensin system (RAS) [15].

TRP metabolism plays a crucial function in blood pressure regulation. Supplementation with TRP has been shown to prevent significant increases in blood pressure and lower blood pressure in patients with primary HTN. Angiotensin-converting enzyme 2 (ACE2) is involved in TRP absorption, and studies using ACE2 knockout rats have revealed a gender-specific difference in blood pressure regulation. In salt-sensitive rats, females lacking ACE2 in the gut microbiota exhibited better protection against HTN-related factors compared to males. This suggests a novel gender-dependent mechanism in blood pressure regulation via the TRP-indole pathway, where salt intake influences TRP metabolism between the host and gut microbiota in a sex-specific manner. These findings emphasize the importance of considering gender-based approaches in the treatment of HTN [70].

#### 3.1.2. AS

AS is a disease characterized by the accumulation of plaques within the arterial walls, leading to their thickening, and is widely regarded as the primary cause of cardiovascular disease [62]. Chronic inflammation, dyslipidemia, and gut microbiota dysbiosis are considered key contributors to the pathogenesis of AS. A fecal metagenomic association study comparing patients with atherosclerotic cardiovascular disease to healthy controls revealed that AS patients exhibited a relative decrease in the abundance of *Bacteroides* and *Prevotella*, alongside a relative increase in *Enterobacteriaceae* and *Streptococcus* compared to healthy individuals. Additionally, an increase in the abundance of oral cavity-associated bacteria, such as *Lactobacillus salivarius* and *Solobacterium moorei*, was observed [71].

As essential amino acids, BCAAs play an indispensable role in maintaining cardiovascular health. However, studies have shown that excessive dietary intake or metabolic disturbances leading to elevated BCAA levels significantly increase plaque volume in the arteries and accelerate the progression of AS. This occurs through the enhanced production of mitochondrial hydrogen peroxide, which promotes macrophage activation and the secretion of disulfide-linked high-mobility group box 1 (HMGB1). HMGB1, in turn, activates the Toll-like receptor (TLR)/nuclear factor kappa-B (NF-κB) pathway, triggering downstream inflammatory cascades that drive the progression of AS [72]. Qiao et al. [73] demonstrated that feeding *Parabacteroides merdae* to high-fat diet (HFD)-induced ApoE^−/−^ mice enhanced the catabolism of BCAAs in the gut and inhibited the mechanistic target of rapamycin complex 1 (mTORC1) pathway in arterial plaques, leading to improved AS in these mice. These findings suggest that modulating BCAA metabolism and limiting excessive BCAA intake may represent an effective strategy for preventing and alleviating AS.

TMAO is considered the “first potential direct link between the gut microbiota and atherosclerotic CVD” [74]. TMAO can increase the expression of receptors such as cluster of differentiation 36 (CD36) and scavenger receptor A1 (SR-A1) in the body, leading to the transformation of macrophages in the arterial wall into foam cells, a process that accelerates plaque formation [75], a key step in AS. TMAO has been found to increase CVD risk by enhancing platelet reactivity and promoting thrombosis, with elevated TMAO levels being independently associated with the risk of thrombotic events in subjects [76]. Additionally, TMAO has been shown to promote the expression of inflammatory genes through the activation of NF-κB and mitogen-activated protein kinase (MAPK) signaling pathways, as well as the nucleotide-binding oligomerization domain-like receptor protein 3 (NLRP3) inflammasome, thereby promoting the development of AS [77]. Furthermore, large-scale clinical studies involving more than 4000 participants have highlighted the potential clinical significance of specifically inhibiting the gut microbiota-derived TMAO pathway in alleviating CVD [76].

#### 3.1.3. HF

HF is a long-term, progressive illness marked by a stiffened or damaged myocardium that prevents the heart from pumping enough blood to meet the body’s metabolic needs. Common clinical manifestations include exercise intolerance, dyspnea, and peripheral edema [78]. Increasing evidence suggests a significant role of the gut microbiota and its metabolites in the pathogenesis and progression of HF. The “gut-heart hypothesis” proposes that in HF, compromised intestinal blood flow leads to ischemia, hypoxia, congestion, and edema of the intestinal mucosa. These alterations disrupt the gut microbiota homeostasis, impair the intestinal barrier, and increase permeability, allowing gut bacteria and microbial metabolites into the bloodstream. This triggers the release of pro-inflammatory cytokines, contributing to chronic systemic inflammation, which further inhibits myocardial contractility, promotes myocardial cell apoptosis and necrosis, and accelerates the progression of HF. A Mendelian randomisation study found six microbial taxa suggestive of a causal relationship with HF, the most significant taxonomic unit being the Bacteroides dorei species [79]. Studies have shown a reduction in microbial diversity in HF patients, with an enrichment of *Ruminococcus gnavus* and a depletion of *Faecalibacterium prausnitzii* in the gut microbiota of HF patients compared to healthy controls [80].

Sustained activation of the sympathetic nervous system in HF patients is considered a major contributor to poor prognosis. In cultured HF-related phenotypes, PAGln has been shown to directly reduce myocardial cell contractility and decrease the expression of B-type natriuretic peptide genes in myocytes and atrial tissue from mice. Further experiments indicate that PAGln attenuates the positive inotropic effects of adrenergic stimulation through interacting with adrenergic receptors [81]. To explore the potential link between PAGln and CVD, both in vivo and in vitro experiments have assessed the impact of PAGln on cardiovascular and cellular functions. Notably, PAGln was found to dose-dependently increase calcium ion concentrations in platelets induced by thrombin [49]. Furthermore, β-blockers, such as carvedilol, were shown to reverse the thrombogenic phenotype induced by PAGln, mitigating its detrimental effects on CVD-related phenotypes at physiological levels, thus providing further evidence of the association between PAGln and CVD [49,82].

A study in DOCA-salt-induced hypertensive mice demonstrated that acetate supplementation reduced kidney fibrosis, improved kidney function, and attenuated cardiac fibrosis and left ventricular hypertrophy. Additionally, it enhanced cardiac structure and function, downregulated early growth response protein 1 in both the heart and kidneys, and modulated the RAS and MAPK signaling pathways to lower blood pressure [83]. Moreover, elevated levels of TMAO have been linked to poor prognosis in HF patients. In HF patients following myocardial infarction, elevated TMAO levels are associated with a worse prognosis. TMAO levels correlate with reduced left ventricular ejection fraction (LVEF) and glomerular filtration rate (GFR), further supporting its role as a potential biomarker for assessing HF severity [84].

### 3.2. Metabolic Disease

**Figure 3:**
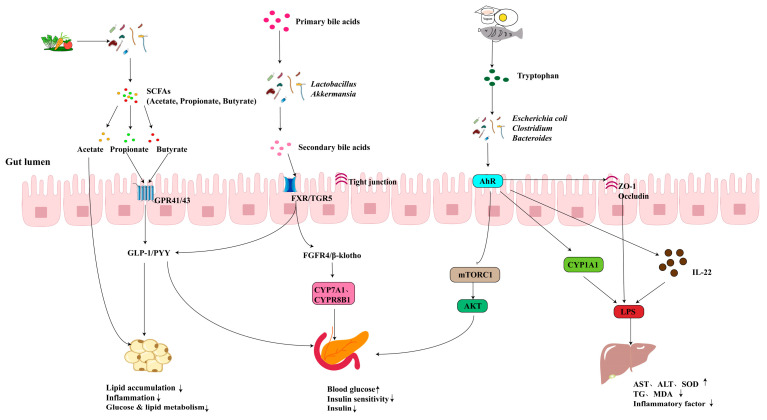
Mechanisms of action of gut microbes and their metabolites in ameliorating metabolic diseases.

Metabolic diseases are a group of disorders caused by abnormalities in one or more components of metabolism, represented by conditions such as obesity, type 2 diabetes (T2D), and metabolic dysfunction-associated steatotic liver disease (MASLD) [85]. In recent years, the global incidence of metabolic diseases has been steadily increasing, posing a significant public health challenge. Genetic predisposition, poor lifestyle, and unhealthy dietary habits are currently recognized as primary risk factors contributing to the development of these conditions [86]. Research has revealed that metabolic disorders are closely associated with alterations in the composition and function of the gut microbiota. Specific microbial-derived metabolites, including BAs, SCFAs, BCAAs, and TRP, have been implicated in the pathogenesis of metabolic diseases (Figure 3). The gut microbiota and its metabolites can influence insulin sensitivity and improve metabolic diseases by modulating energy balance as well as lipid and glucose metabolism [7].

#### 3.2.1. Obesity

Obesity is characterized by abnormal or excessive fat accumulation and is closely related to an imbalance between energy intake and expenditure. With the rising global prevalence of obesity, it has become a major public health issue. Early studies have shown that obesity is associated with a significant reduction in the diversity of the gut microbiota [87]. Dysbiosis of the gut microbiota may contribute to obesity through mechanisms involving disrupted energy metabolism, enhanced fat synthesis and storage, dysregulated appetite control, and chronic low-grade inflammation. These interconnected pathways establish a causal link between alterations in gut microbial composition and obesity pathogenesis. Notably, depletion of the gut microbiota in obese mice with antibiotics reversed weight gain and leptin resistance, thus emphasizing the pathogenic role of the microbiota.

Ke et al. demonstrated that an increased abundance of SCFA-producing bacteria such as *Akkermansia*, *Alistipes*, *Bacteroides*, and *Phascolarctobacterium* can significantly reduce body weight gain and excessive fat accumulation in high-fat diet-fed mice, as well as alter the expression of key genes involved in lipid metabolism [88,89]. SCFAs influence gut barrier function and the microbiota composition, reducing inflammation and intestinal permeability, which in turn impacts host metabolic health [90,91]. Additionally, SCFAs can directly activate G protein-coupled receptors such as GPR43 and GPR41 to improve metabolic diseases. GPR43 is primarily expressed in white adipose tissue and can directly inhibit insulin signaling in adipocytes, thereby reducing fat accumulation in adipose tissue and promoting lipid and glucose metabolism in other tissues. GPR41, an energy sensor located in the sympathetic nervous system and gut, is activated by SCFAs like propionate and butyrate. When activated, GPR41 stimulates enteroendocrine cells to secrete peptide YY (PYY), a hormone that promotes satiety and helps regulate appetite [92,93,94].

Furthermore, research has shown that TRP metabolites, such as indole-3-propionic acid (IPA) and indole, can activate the aryl hydrocarbon receptor (AHR) pathway, significantly increasing the expression of interleukin-22 (IL-22) and glucagon-like peptide-1 (GLP-1). These metabolites upregulate the expression of tight junction proteins, antimicrobial peptides, and mucins to reduce lipopolysaccharides, thereby improving gut barrier function. Additionally, TRP metabolites activate the IL-22R/signal transducer and activator of transcription 3/acyl-CoA oxidase 1 signaling pathway, enhancing lipid metabolism, and the GLP-1R/phospho -cAMP-response element binding protein pathway, which improves insulin resistance [95]. A study investigating the protective effects of a ketogenic diet (KD) against obesity and related complications identified taurodeoxycholic acid (TDCA) and tauroursodeoxycholic acid (TUDCA) as key endogenous metabolites contributing to the anti-obesity effects of a KD. A KD reduces the abundance of *Lactobacillus murinus* ASF361, a gut microbe that encodes bile salt hydrolase, leading to decreased uncoupling of TDCA and TUDCA and increased circulating levels of these metabolites. This, in turn, reduces energy absorption by inhibiting the expression of intestinal carbonic anhydrase 1, ultimately promoting weight loss. Furthermore, TDCA and TUDCA independently exert anti-obesity effects, offering promising new therapeutic targets for the treatment of obesity and its associated complications [25].

#### 3.2.2. T2D

T2D is the most common metabolic disease and has become a global epidemic, and the hyperglycemia is a hallmark of T2D, caused by insufficient insulin secretion. Therefore, the core of T2D treatment is to address the issue of hyperglycemia [96]. Energy balance disruption caused by gut microbiota dysbiosis plays an indispensable role in the progression of T2D. Increasing attention is being paid to altering the gut microbiota composition to enhance insulin sensitivity, and gut microbiota therapy has emerged as a novel therapeutic approach [97]. Research has indicated that the gut microbiota composition of people with T2D varies considerably from that of people with normal blood glucose levels. Specifically, the abundance of Clostridia is significantly reduced, while the abundance of *Sutterella* and *Streptococcus* is significantly increased. Further studies have found that genera such as *Ruminococcus*, *Clostridium*, and *Desulfovibrio* promote the development of T2D, while genera like *Bifidobacterium*, *Bacteroides*, and *Akkermansia* can inhibit the progression of T2D [98].

SCFAs, as metabolic products of the gut microbiota, play multiple roles in controlling immune regulation, regulating insulin secretion, and promoting pancreatic β-cell proliferation in insulin resistance and T2D [99]. Studies have shown that butyrate can induce the secretion of GLP-1 and PYY, increasing energy expenditure and maintaining glucose homeostasis [19]. Dysfunction and loss of β-cells are characteristic features of T2D. Recent studies have found that elevated levels of TMAO impair glucose-stimulated insulin secretion and reduce the proportion of β-cells in mice, as well as impair glucose tolerance. However, when the FMO3 gene is knocked out in mice, plasma TMAO levels are significantly reduced, and improvements in pancreatic morphology, β-cell function, and glucose tolerance are observed. Therefore, knocking out FMO3 and inhibiting TMAO may be an effective strategy for treating T2D [12].

#### 3.2.3. MASLD

Non-alcoholic fatty liver disease (NAFLD) is a globally prevalent chronic liver disorder characterized by excessive hepatic fat accumulation, inflammation, hepatocellular injury, and fibrosis. These pathological conditions range from hepatic steatosis to non-alcoholic steatohepatitis (NASH), and further progress to cirrhosis and hepatocellular carcinoma [100]. MASLD, a term introduced by Eslam et al. in 2020, refers to fatty liver disease associated with metabolic syndrome [101,102]. Overweight/obesity, T2D, and metabolic dysfunction are key diagnostic criteria for MASLD [101]. A recent study demonstrated that time-restricted feeding significantly enriched the gut microbiota with *Ruminococcus* torques and, through its microbial metabolite 2-hydroxy-4-methylpentanoic acid, inhibited the intestinal Hypoxia-inducible factor-2alpha sphingolipid pathway, alleviating inflammation and fibrosis in a mouse model of metabolic dysfunction-associated steatohepatitis, highlighting the beneficial role of R. torques and its metabolites in MASLD [103].

Recent evidence underscores the crucial role of SCFAs in the pathogenesis of MASLD. SCFAs influence hepatic lipid metabolism by regulating fatty acid oxidation and synthesis in the liver. For example, acetate inhibits the expression of fatty acid synthase (FASN) and CD36, thereby modulating liver fat metabolism [20]. Additionally, SCFAs exhibit anti-inflammatory effects by suppressing the NF-κB signaling pathway, reducing the expression of inflammatory cytokines, and alleviating liver inflammation in MASLD [104]. SCFAs also enhance gut barrier function, reduce bacterial translocation and endotoxin leakage, and further decrease liver inflammation [105]. In patients with hepatic steatosis, fecal levels of IPA and indole-2-acetic acid (IAA) are significantly reduced compared to healthy individuals. Exogenous supplementation of IPA and IAA has been shown to lower endotoxin levels, inhibit macrophage activation, and suppress the NF-κB signaling pathway, thereby improving liver steatosis and inflammation in a Western diet-induced MASLD mouse model [106]. Thus, modulating the gut microbiota and its metabolic derivatives represents a promising therapeutic strategy for MASLD.

### 3.3. Gastrointestinal Diseases

Inflammatory bowel disease (IBD), encompassing Crohn’s disease (CD) and ulcerative colitis (UC), is a chronic, recurrent autoimmune inflammatory disease that affects the gastrointestinal tract and extra-intestinal organs [107]. Over the past two to four decades, IBD’s incidence and prevalence have grown globally, with its pathogenesis being multifactorial and complex. Genetic susceptibility, environmental factors, intestinal barrier dysfunction, and immune responses are key contributors [108]. Furthermore, IBD pathogenesis is closely associated with gut microbiota dysbiosis, characterized by reduced microbial diversity and a disrupted balance between commensal and pathogenic bacteria. A clinical study involving 132 participants over one year revealed that IBD onset coincided with gut microbiota dysbiosis [109]. Regulatory strategies aimed at restoring gut microbiota homeostasis have thus become an important approach in IBD treatment. Studies show that, compared to healthy individuals, IBD patients exhibit reduced abundance of *Faecalibacterium prausnitzii*, while the abundance of *Ruminococcus gnavus*, *Bacteroides fragilis*, *Escherichia coli*, and *Clostridium innocuum* is increased. These bacteria promote inflammation by producing pro-inflammatory polysaccharides and disrupting the intestinal mucosal barrier [107].

**Figure 4:**
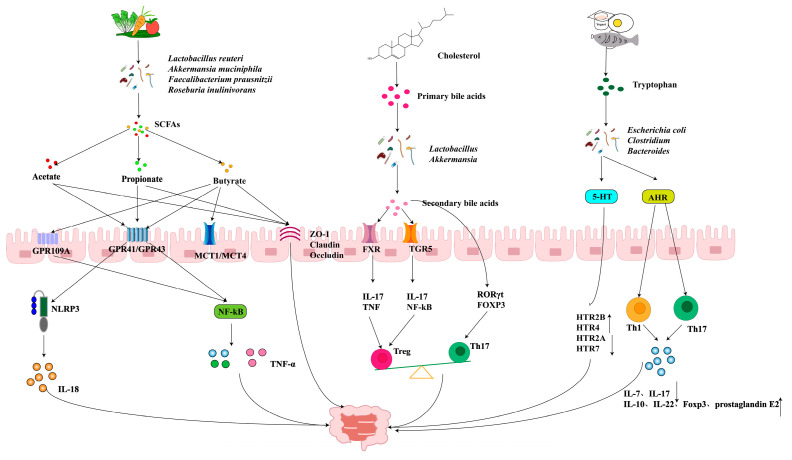
Mechanisms of action of intestinal microorganisms and their metabolites in ameliorating gastrointestinal diseases.

In an untargeted metabolomics and shotgun metagenomics analysis of fecal samples from 155 individuals with CD, UC, and 65 non-IBD controls, IBD patients were found to have lower levels of key metabolites, highlighting the strong association between gut microbiota, its metabolites, and IBD [110]. Specifically, gut microbiota-derived metabolites, particularly BAs, SCFAs and TRP metabolites, have emerged as potential biomarkers for diagnosing and predicting IBD (Figure 4).

#### 3.3.1. BAs and IBD

Increasing evidence underscores the pivotal roles of the gut microbiota and BAs in maintaining gut homeostasis and modulating inflammation. Dysbiosis and BAs metabolic disorders impair the intestinal barrier and immune function [111]. In a study investigating the effects of dihydro-berberine in UC, it was shown that increasing the abundance of BA receptor agonists, such as CDCA and lithocholic acid, activated the FXR/TGR5 signaling pathways in intestinal epithelial cells, exerting anti-inflammatory effects and promoting intestinal barrier repair [28]. Furthermore, the imbalance between T-helper 17 (Th17) and regulatory T (Treg) cells is a key factor in IBD. Th17 cells drive tissue inflammation, while Treg cells suppress autoimmunity. In IBD patients, gut microbes associated with the Th17/Treg balance, such as *Ligilactobacillus*, *Lactobacillus*, *Bacteroides*, and *Akkermansia*, along with microbial metabolites like BAs, are significantly enriched, indicating that BAs may regulate the Th17/Treg balance [112]. A recent experimental study found that short-term high-fat diets exacerbate colitis through tumor necrosis factor (TNF)-mediated bile acid tolerance impairment, suggesting that BAs act as “opportunistic pathogens” in the gut. Thus, modulating BAs balance may provide a promising target for the IBD prevention and treatment [113].

#### 3.3.2. SCFAs in IBD

SCFAs are an important energy source for colonic mucosal cells and are essential metabolites for maintaining intestinal homeostasis. They play a protective role in patients with IBD [21]. A comparison of fecal SCFA concentrations between 34 IBD patients and 30 healthy controls revealed that the SCFA levels in the feces of IBD patients were generally lower than those of the healthy controls [114]. In both in vivo and in vitro studies, SCFAs, particularly butyrate, have been shown to inhibit neutrophil production of pro-inflammatory cytokines and chemokines in IBD. Additionally, butyrate inhibits histone deacetylase (HDAC) activity, neutrophil migration, and the formation of neutrophil extracellular traps, which improves DSS-induced colitis in mice [115]. GPR109A, a G protein-coupled receptor activated by butyrate, plays a crucial role in suppressing inflammation in various diseases. In colitis models induced by 2,4,6-trinitrobenzenesulfonic acid in GPR109A^−/−^ and wild-type mice, butyrate significantly ameliorated the inflammatory response and intestinal epithelial barrier dysfunction in wild-type mice, but had no effect in GPR109A^−/−^ mice. In addition, in vivo experiments showed that butyrate could inhibit the phosphorylation of NF-κB and protein kinase B signaling pathways, improving the inflammatory response [116]. Additionally, SCFAs may inhibit histone deacetylases HDAC1 or HDAC3, suppressing interleukin 17 (IL-17) production by γδT cells in the colon and cecum, enhancing our understanding of γδT cells regulation and offering new therapeutic avenues for IBD [117]. In IBD patients, the abundance of SCFA-producing bacteria is significantly reduced, leading to lower SCFA levels and increased intestinal inflammation. Therefore, restoring gut microbiota balance and supplementing SCFAs may represent effective strategies for IBD treatment.

#### 3.3.3. AHR and Its Role in IBD

AHR is a ligand-activated transcription factor, and clinical findings have shown that AHR expression is lower in IBD patients compared to healthy intestinal epithelial cells, where its expression and activation are elevated [118]. AHR plays a crucial role in regulating the differentiation and activity of Th1 and Th17 cell populations. Its activation reduces the expression of inflammatory cytokines such as IL-7 and IL-17, while increasing the levels of IL-10, IL-22, prostaglandin E2, and forkhead box p3 (Foxp3), thereby modulating the inflammatory response under inflammatory conditions [119]. Consequently, modulating AHR has been considered a potential therapeutic target for treating IBD. TRP can be converted by the gut microbiota into bioactive indole metabolites, which are potent agonists of AHR [120]. Zinc plays an essential role in maintaining gut development and intestinal mucosal barrier function, and zinc supplementation is widely used in the treatment of diarrhea and other related gastrointestinal diseases. Research by Zhou et al. demonstrated that indole-3-carbinol activates AHR, upregulating the transcriptional activity of zinc transporter proteins, increasing cellular total zinc and cytosolic free Zn^2+^ concentrations, and subsequently inhibiting NF-κB signaling and the degradation of tight junction proteins. This promotes the expression of intestinal mucosal tight junction proteins in a zinc-dependent manner, enhancing intestinal barrier function [121].

Additionally, a recent study found that AHR deficiency promotes macrophage pyroptosis and the secretion of inflammatory cytokines such as IL-1β. Activation of AHR binds to the promoter region of ornithine decarboxylase (ODC1), inducing ODC1 transcription and producing polyamine metabolites that suppress macrophage pyroptosis and ameliorate IBD symptoms [122]. Indole metabolites such as indole-3-lactic acid (ILA), IPA, and IAA interact with gut microbiota to enhance the abundance of TRP-metabolizing bacteria (e.g., *Clostridium*) and the expression of enzymes such as acyl-CoA dehydrogenase, which boosts the production of IPA and IAA, alleviating intestinal inflammation [123]. In IBD patients exacerbated by a HFD, IAA levels are reduced, but supplementation of IAA can upregulate the expression of sulfotransferase enzymes such as 3-phosphoadenosine 5-phosphosulfate synthase and solute carrier family 35 member B3 (Slc35b3), thereby enhancing intestinal mucin sulfation and protecting gut homeostasis, which effectively relieves colitis [58]. Furthermore, studies have shown that TRP can reduce the production of lipopolysaccharide-binding protein in the gut and regulate macrophage activation, alleviating intestinal inflammation. Experimental evidence also suggests that TRP metabolites, such as indole, can activate the 5-hydroxytryptamine receptor 2B (HTR2B) receptor, reducing LBP production and polarization of M1 macrophages, thereby alleviating DSS-induced colitis [124].

However, in IBD patients, reduced gut microbiota-mediated TRP metabolism leads to disturbances in the intestinal microenvironment and the development of intestinal inflammation. Therefore, promoting TRP catabolism is crucial for improving IBD.

Additionally, a recent study on newly diagnosed CD patients found a strong correlation between dietary-derived PAGln and CD, suggesting it may exacerbate colitis in mouse models via platelet activation and could serve as an early diagnostic marker for CD [125]. Outer membrane vesicles (OMVs) are double-membrane nanostructures containing nucleic acids, proteins, lipopolysaccharides, and other components, typically released by Gram-negative bacteria such as *Akkermansia muciniphila* during growth. Transplantation of OMVs, rather than live bacteria, into the mouse gut alleviates colitis and enhances the efficacy of anti-programmed cell death protein immunotherapy in CRC treatment. This suggests OMVs could be a novel therapeutic target, potentially restoring gut microbiota balance and maintaining intestinal barrier integrity [126].

We have compiled the changes in gut microbes and their metabolites in different diseases, as shown in Table 2. We found that gut microbes and their metabolites exhibit both synergistic and antagonistic roles in a variety of chronic diseases by modulating host metabolic, immune, and inflammatory responses. Decreased SCFAs, a common feature across diseases, exacerbate inflammation and impaired energy metabolism in cardiovascular diseases such as hypertension, atherosclerosis, and heart failure; inhibit insulin sensitivity in obesity, T2D, and MASLD; and lead to impaired intestinal barrier function in CD and UC, whereas elevated TMAO, by promoting thrombosis and foam cell accumulation, generally exacerbates cardiovascular disease risk. Metabolic imbalances in BAs are bi-directionally regulated in different diseases: reduced bound BAs in obese patients impair FXR signaling, whereas elevated secondary BAs in MASLD and CD drive hepatic lipid synthesis and intestinal Th17 inflammation, respectively. Changes in the abundance of specific genera such as *Faecalibacterium prausnitzii* and *Akkermansia muciniphila*, on the other hand, are disease-specific, with the former reduction exacerbating metabolic disorders in HF and intestinal inflammation, and the latter deletion exacerbating insulin resistance in obesity and diabetes. In addition, the metabolic paradox of BCAAs suggests a complex regulatory network in different diseases. These findings suggest that targeting SCFAs supplementation, TMAO production inhibition, or colony dynamic balance modulation may be a cross-disease intervention strategy, but the therapeutic pathway needs to be optimized in the context of the disease in order to achieve precise regulation of the ‘colony–metabolite–host’ network.

## 4. Targeted “Gut Microbiota” Therapies

**Figure 5:**
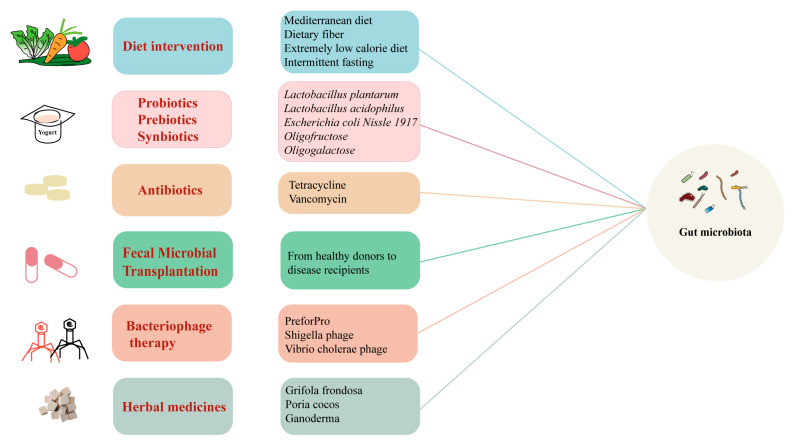
Schematic of new therapeutic approaches targeting the “gut microbiota” for treating chronic diseases.

Increasing evidence highlights the significant role of the gut microbiota in chronic diseases, indicating that modifying its composition could prevent or ameliorate these conditions. Strategies to target the gut microbiota include dietary interventions, probiotics, prebiotics, synbiotics, antibiotics, and FMT (Figure 5).

### 4.1. Dietary Intervention

Diet is a major determinant of health and a key contributor to chronic non-communicable diseases. With the growing prevalence of Western diets, the incidence of chronic diseases continues to rise, highlighting the profound impact of diet on health. The composition of food can affect the gut microbiota’s composition and function, thereby influencing overall health. Dietary interventions have been proven to be an effective strategy for reducing the risk of chronic diseases. Dietary changes account for 57% of variations in the gut microbiota, while host genetics contribute less than 12% [144]. The “Western diet”, characterized by low fiber and high fat and carbohydrate intake, is a major factor leading to gut microbiota dysbiosis. In contrast, the “Mediterranean” and vegetarian diets, which are rich in fruits, vegetables, and dietary fibers, can help prevent dysbiosis. Dietary fiber, found in fruits, vegetables, grains, legumes, and nuts, serves as a key nutrient for gut microbiota, promoting the production of SCFAs that benefit host health. A one-year Mediterranean diet intervention in elderly individuals showed that long-term adherence to this diet positively changed the gut microbiota, improved cognitive function, and reduced inflammation, demonstrating the feasibility of dietary changes to modulate the gut microbiota [145]. Similarly, a very low-calorie diet study in obese patients altered the gut microbiota composition, particularly increasing the abundance of *Bacteroides*. Additionally, intermittent fasting in rats increased gut microbiota α-diversity and reduced the *Firmicutes*/*Bacteroidetes* ratio [146]. The interactions between diet, the gut microbiota, and host health are complex and influence metabolic and immune responses.

### 4.2. Probiotic, Prebiotic, and Synbiotic Interventions

Probiotics are live microorganisms that provide health benefits to the host by directly acting on the gut microbiota, improving microbial balance, enhancing gut barrier function, and modulating immune responses [147]. Specific probiotics such as *Lactobacillus plantarum* and *Lactobacillus rhamnosus* GR-1 have been shown to alleviate HF and myocardial infarction in animal models [148]. In IBD patients, TNF-α significantly increases intestinal permeability. Studies have shown that in intestinal epithelial cells, the probiotic Lactobacillus acidophilus protects intestinal epithelial tight junction barrier integrity by inhibiting TLR2-dependent NF-κB p50/p65 activation, thereby preventing DSS-induced colitis [149]. Overexpression of catalase and superoxide dismutase in *Escherichia coli* Nissle 1917, encapsulated in chitosan/alginate, administered to various chemically induced IBD mouse models, effectively alleviates inflammation, and repairs the colonic epithelial barrier. This suggests that probiotic supplementation can alleviate intestinal inflammation, regulate the gut microbiota, and treat gut-related diseases [150].

Prebiotics, such as oligosaccharides (e.g., fructooligosaccharides, galactooligosaccharides, inulin), are dietary components selectively utilized by beneficial gut microbes to confer health benefits by promoting the growth and activity of beneficial bacteria, indirectly impacting host health [147]. Prebiotics stimulate the fermentation of beneficial gut bacteria, producing SCFAs, which provide energy to intestinal epithelial cells, regulate lipid metabolism, and stimulate the production of anti-inflammatory cytokines, thereby maintaining intestinal epithelial homeostasis and positively affecting chronic inflammatory diseases [21]. In 2019, the International Scientific Association for Probiotics and Prebiotics updated the definition of synbiotics as “a mixture of live microorganisms and substrates selectively utilized by the host’s microbiota that confer health benefits to the host” [151]. A meta-analysis of 3835 T2D patients demonstrated that probiotics, prebiotics, and synbiotics significantly decreased fasting blood glucose and insulin levels [152]. Moreover, clinical trials have shown that synbiotic therapy can reduce colorectal proliferation, improve epithelial barrier integrity and function, and lower the risk of colorectal cancer (CRC) [153]. These findings underscore the potential of probiotic, prebiotic, and synbiotic therapies in the management of chronic diseases, offering new strategies for prevention and treatment. However, further research is required to optimize the administration, dosages, and efficacy of these interventions.

### 4.3. Antibiotics

The primary aim of antibiotic interventions is to eliminate pathogenic microbiota. However, with the widespread use of antibiotics, antibiotic resistance has become a growing issue. In 2023, only one new antibiotic was approved by the Food and Drug Administration, highlighting the slow pace of new antibiotic development compared to the rapid development of resistance [154]. Antibiotic therapy is a non-specific antimicrobial approach that not only targets pathogenic microbes but also disrupts the beneficial microbiota essential for maintaining host health. Experimental studies in adolescent/post-adolescent C58BL/6T mice exposed to tetracycline during puberty have revealed sustained alterations in the gut microbiota, along with disruptions in the gut FXR-FGF15 axis. These changes are associated with liver metabolic dysregulation and increased fat accumulation in subcutaneous, visceral, and skeletal muscle tissues [155]. While antibiotics are effective in eradicating pathogenic organisms, they also deplete beneficial microbes, potentially compromising gut health. Current research is focused on optimizing antibiotic treatment regimens, including appropriate duration and dosage, to minimize resistance development and improve clinical outcomes. Additionally, further studies are needed to assess the long-term safety and efficacy of antibiotics, particularly in the context of microbiota disruption.

### 4.4. FMT and Phage Therapy

FMT has emerged as an important intervention for restoring gut microbiota dysbiosis, involving transferring fecal material from a healthy donor into the recipient’s gut to re-establish a healthy microbiota [156]. FMT has been widely accepted as an effective treatment for persistent or refractory Clostridium difficile infections [157]. A 12-week, double-blind, randomized, placebo-controlled trial of FMT capsules demonstrated metabolic improvements in patients, including increased insulin sensitivity, as measured by the hyperinsulinemic-euglycemic clamp technique. Fecal microbiota sequencing using 16S rRNA technology revealed significant changes in the microbiota composition and weight loss in the FMT group [158]. The gut virome, primarily composed of bacteriophages, attacks bacteria in a host-specific manner and has the potential to alter the gut microbiota. Research has shown that transplanting the fecal virome from lean donor mice to obese mice resulted in reduced weight gain and normalized blood glucose levels [159]. While short-term FMT treatment is generally considered safe and effective, the long-term consequences of altering the gut microbiota remain unclear. Potential risks of FMT include the transmission of pathogens, gastrointestinal symptoms such as diarrhea and abdominal discomfort, and the lack of established safety in immunocompromised individuals. Long-term follow-up studies are essential to evaluate the durability of FMT effects and to identify any potential adverse outcomes. Future research will likely focus on the development of targeted microbiota transplantation strategies, emphasizing functional strains that can be tailored to treat specific diseases, thus enabling precision therapy.

## 5. Future Outlook

The composition of the gut microbiota and its derived metabolites play a pivotal role in human health and disease. Emerging research underscores the gut microbiota’s influence on host metabolic pathways, immune responses, and inflammation, which are directly or indirectly implicated in the onset and progression of chronic diseases. In CVD, the gut microbiota produces metabolites such as SCFAs, TMAO, and BAs, which influence vascular health and blood pressure regulation, potentially improving diseases such as AS and HTN. In metabolic diseases, the gut microbiota regulates energy balance and lipid/glucose metabolism, modulates insulin sensitivity, and is closely associated with obesity, T2D, and MASLD. In gastrointestinal diseases, the gut microbiota maintains intestinal barrier integrity, enhances gut permeability, and reduces the infiltration of inflammatory factors, thus alleviating intestinal inflammation.

Regulating the gut microbiota’s homeostasis provides novel therapeutic strategies for chronic diseases management. Personalized dietary interventions, particularly increasing fiber intake, can promote beneficial bacterial growth, improve microbiota balance, and positively impact chronic diseases. Supplementing with probiotics and prebiotics can modulate the microbiota composition, enhance intestinal barrier function, and reduce inflammation, offering therapeutic potential for managing chronic conditions. FMT, an emerging therapeutic strategy, has shown promise in treating various chronic diseases by restoring a healthy microbiota. Additionally, certain drugs, such as antibiotics, have been found to alter the composition of the gut microbiota, highlighting microbiota modulation as a potential treatment strategy for chronic diseases.

Early intervention is crucial for the prevention and management of chronic diseases. For individuals at risk of chronic diseases, such as those with prehypertension, early intervention through gut microbiota modulation may help delay or prevent the onset of chronic diseases. Managing chronic diseases requires a multifaceted approach that incorporates genetic, environmental, lifestyle, and microbiota factors. Regulating the gut microbiota offers a new dimension in chronic disease therapy.

The relationship between the gut microbiota and chronic diseases is complex and profound, and regulating the microbiota presents a new avenue for disease prevention and treatment. Current research on the gut microbiota is advancing rapidly, with emerging artificial intelligence tools used to identify the core microbiome associated with human health. These approaches leverage metagenomic sequencing to accurately define the core microbiome, providing new targets for precision medicine. This progress presents exciting opportunities for personalized treatments for chronic diseases related to microbiota imbalances [160]. However, many questions remain unanswered, particularly regarding whether observed changes in the microbiota are causative or merely a consequence of disease. Additionally, further research is needed to understand how the gut microbiota interacts with the host’s genetic and environmental factors, and how these interactions influence disease progression. As research continues to evolve, we are likely to see more microbiota-based therapeutic options, offering more effective treatments for patients with chronic diseases.

## Figures and Tables

**Table 1:** Study summarizes link between gut microbiota metabolites and chronic disease.

Metabolites	Disease	Mechanisms	References
TMAO	Hypertension	Promoting Ang II-induced vasoconstriction and thus Ang II-induced hypertension	[8]
	Atherosclerosis	Increased expression of CD36 and SRA causes cholesterol to accumulate in cells, promotes macrophage foaming, and accelerates atherosclerotic plaque formation	[9]
	Heart failure	Enhancing NADPH oxidase NOX activity and increases reactive oxygen species ROS production, further damaging cardiomyocytes and promoting pathological remodeling	[10]
	Obesity	Increased visceral fat	[11]
	Type 2 diabetes	Reducing the proportion of beta cells and glucose tolerance in mice with impaired insulin secretion	[12]
	Metabolic dysfunction-associated steatotic liver disease	Promotes lipid deposition in HepG2 fatty liver cells, disrupting the structure and function of the intestinal barrier and exacerbating hepatic steatosis	[13]
	Inflammatory bowel disease	Involvement in IBD pathogenesis by affecting ATG16L1-induced autophagy and activation of NLRP3 inflammasome	[14]
SCFAs	Hypertension	Regulation of microbiome composition, increased expression of SCFA receptors GPR41 and GPR109A, and restoration of RAS homeostasis in kidney	[15]
	Atherosclerosis	Reduces inflammation, improves metabolic health, and stabilizes plaque	[16]
	Heart failure	Improved left ventricular remodeling in mice	[17]
	Obesity	Upregulation of UCP1 (a key protein involved in energy expenditure) expression increases white fat browning and energy expenditure	[18]
	Type 2 diabetes	Induction of GLP-1 and PYY secretion increases energy expenditure and maintains glucose homeostasis	[19]
	Metabolic dysfunction-associated steatotic liver disease	Inhibition of hepatic FASN and CD36 protein expression regulates hepatic lipid metabolism	[20]
	Inflammatory bowel disease	Maintaining intestinal homeostasis, enhancing intestinal barrier function, controlling intestinal inflammation	[21]
BAs	Hypertension	Activation of TGR5 in neurons and microglia attenuates inflammatory responses and oxidative stress, inhibits activated neurons, and attenuates hypertension	[22]
	Atherosclerosis	Activation of TGR5, reduction in macrophage inflammation and lipid uptake, inhibition of platelet activation	[23]
	Heart failure	Reduces pro-inflammatory factors and improves peripheral blood flow	[24]
	Obesity	Reduces energy absorption by inhibiting intestinal Car1 expression, leading to weight loss	[25]
	Type 2 diabetes	Promote GLP-1 secretion, thus increasing insulin release and lowering blood glucose	[26]
	Metabolic dysfunction-associated steatotic liver disease	Increases TGR5 and FXR signaling, improves metabolic disorders, prevents steatosis and hepatocyte ballooning, and reduces macrophage infiltration	[27]
	Inflammatory bowel disease	Activation of intestinal epithelial FXR/TGR5-related signaling pathway to inhibit inflammatory response and repair intestinal barrier integrity	[28]

**Table 2:** Changes in gut microbes and their metabolites in different diseases.

Disease	Model	Sample Size	Gut Microbiota	Metabolites	References
Hypertension	Human	n = 29	*Escherichia*/*Shigella* ↑ *Tyzerella* 4, *Gordonibacter* and *Fournierella*	Acetic, propionic, and butyric acids ↓	[127]
Hypertension	Human	n = 205	*Odoribacter*, *Clostridiaceae* ↓	Butyrate ↓	[128]
Hypertension	Human	n = 129	*Parabacteroides*, *Desulfovibrio*, *Christensenella*, *Alistipes* ↑ *Prevotella*, *Lactobacillus* ↓	SCFAs ↓, TMAO ↑	[129]
Atherosclerosis	Human	n = 76	*Prevotella copri* ↑	TMAO ↑	[130]
Atherosclerosis	Human	n = 405	*Enterobacteriaceae* and *Streptococcus* spp. ↑ *Bacteroides* and *Prevotella* ↓	/	[71]
Atherosclerosis	Female and male C57BL/6J mice	n = 342	*Roseburia* ↓	Butyrate ↓	[131]
Heart Failure	Human	n = 94	*Faecalibacterium prausnitzii* ↓ and *Ruminococcus gnavus* ↑	Butyrate ↓ TMAO ↑	[80]
Heart Failure	Human	n = 24	*Eubacterium rectale* and *Dorea longicatena* ↓	Butyrate, acetate ↓	[124]
Heart Failure	Human	n = 20	*Blautia*, *Collinsella*, uncl. *Erysipelotrichaceae* and uncl. *Ruminococcaceae* ↓	/	[132]
Obesity	Human	n = 134	*Lactobacillus*, *Akkermansia*, *Christensenellaceae*, *Methanobrevibacter* ↓ *Paraprevotella* ↑	Bile acids, glycocholic acid, glycoursodeoxycholic acid, taurohyodeoxycholic acid, and tauroursodeoxycholic acid ↑	[133]
Obesity	Human	n = 32	*Bacteroidetes* ↑ *Firmicutes*, *Verrucomicrobia* ↓	Arginine, glutamine, 2-oxoisovalerate, pyruvate, alanine ↑; citrate, BCAA ↓	[134]
Obesity	Human	n = 72	*Eubacterium hallii*, *Ruminococcus gnavus groups*, and *Dorea* ↑	L-isoleucine, uric acid ↑Taurodeoxycholic, tauromuricholic α + β acid, myristic acid ↓	[135]
Type 2 Diabetes	Male Wistar rats	n = 30	*Bacteroidetes*, *Prevotella*, *Deltaproteobacteria*, *Oscillospira*, *Veillonellaceae*, *Phascolarctobacterium*, *Sutterella*, *Bilophila* ↓	Acetic acid, propionic acid, butyric acid, isobutyric acid, valeric acid, isovaleric acid ↓	[136]
Type 2 Diabetes	Male C57BL/6J mice	n = 48	*Bacteroidetes* ↓ *Firmicutes* ↓ *Lactobacillus*, *Akkermansia*, *Bacteroides*, *Bifidobacterium* ↓ *Helicobacter* ↑	Acetic acid, propionic acid, butyric acid, isobutyric acid, valeric acid, isovaleric acid ↓	[137]
Metabolic Dysfunction-Associated Steatotic Liver Disease	Female BALB/c mice	n = 24	*Firmicutes*, *Bacteroidetes*, *Proteobacteria* ↓ *Akkermansia muciniphila*, *Clostridium leptum*, *Ruminococcus gnavus* ↓	Propionic acid, butyric acid ↓	[138]
Metabolic Dysfunction-Associated Steatotic Liver Disease	Male C57BL/6J mice	n = 40	*Alistipes*, *Ruminiclostridium*, *Rikenella* ↓ *Lactococcus*, *Enterorhabdus*, *Turicibacter*, *Clostridium-sensu-stricto-1*, *Tyzzerella*, *Oscillibacter* ↑	Acetic acid, propionic acid, butyric acid, isobutyric acid, valeric acid, isovaleric acid ↓	[139]
Metabolic Dysfunction-Associated Steatotic Liver Disease	Human	n = 61	*Bacteroidetes* ↓, *Bifidobacterium*, *Firmicutes* ↑	Cholic acid, chenodeoxycholic acid ↑	[106]
Crohn’s Disease	Human	n = 220	*Bifidobacterium breve, Clostridium symbiosum* ↑ *Roseburia hominis, Dorea formicigenerans, Ruminococcus obeum* ↓	Cholic acid, deoxycholic acid ↑	[109]
Crohn’s Disease	Human	n = 15	*Anaerostipes, Roseburia, Ruminococcus, Lactobacillus* ↓	Acetic acid, propionic acid, butyric acid ↓	[140]
Crohn’s Disease	Male C57BL/6J mice	/	*Lactobacillus* ↓	purine ↑ uric acid ↓	[141]
Ulcerative Colitis	Human	n = 58	*Enterobacteriaceae*, *Veillonella*, *Streptococcus*, and *Bacteroides* ↑	Hexanoate, butyrate/propionate, LCA and DCA ↓ UDCA ↑	[142]
Ulcerative Colitis	Human	n = 240	*Lactobacillales, Sellimonas, Streptococcus* ↑ *Prevotella_9, Lachnospira* ↓	Glycocholic acid, glycochenodeoxycholic acid ↑	[143]

## Abbreviations

The following abbreviations are used in this manuscript:

CVD	cardiovascular disease
HTN	hypertension
AS	atherosclerosis
HF	heart failure
T2D	type 2 diabetes
MASLD	metabolic dysfunction-associated steatotic liver disease
IBD	inflammatory bowel disease
CRC	colorectal cancer
NCDs	non-communicable diseases
TMAO	trimethylamine N-oxide
TMA	trimethylamine
SCFAs	short-chain fatty acids
BAs	bile acids
PAGln	phenylacetylglutamine
BCAAs	branched-chain amino acids
TRP	tryptophan
FMT	fecal microbiota transplantation
FMOs	flavin-containing monooxygenases
GPRs	G protein-coupled receptors
CDCA	chenodeoxycholic acid
FXR	Farnesoid X Receptor
TGR5	G protein-coupled bile acid receptor
5-HT	serotonin
PVN	paraventricular nucleus
ANG II	angiotensin II
RAS	renin-angiotensin system
ACE2	Angiotensin-converting enzyme 2
HMGB1	high-mobility group box 1
TLR	Toll-like receptor
NF-κB	nuclear factor kappa-B
mTORC1	mechanistic target of rapamycin complex 1
CD36	cluster of differentiation 36
SR-A1	scavenger receptor A1
MAPK	mitogen-activated protein kinase
NLRP3	nucleotide-binding oligomerization domain-like receptor protein 3
LVEF	left ventricular ejection fraction
GFR	glomerular filtration rate
AHR	aryl hydrocarbon receptor
IL-22	interleukin-22
GLP-1	glucagon-like peptide-1
KD	ketogenic diet
TDCA	taurodeoxycholic acid
TUDCA	tauroursodeoxycholic acid
NAFLD	non-alcoholic fatty liver disease
NASH	non-alcoholic steatohepatitis
FASN	fatty acid synthase
IPA	indole-3-propionic acid
IAA	indole-2-acetic acid
CD	Crohn’s disease
UC	ulcerative colitis
CDCA	chenodeoxycholic acid
Th17	T-helper 17
Treg	regulatory T
TNF	tumor necrosis factor
HDAC	histone deacetylase
IL-17	interleukin 17
Foxp3	forkhead box p3
ODC1	ornithine decarboxylase
ILA	indole-3-lactic acid

## References

[1] Yang Z.-Y., Tang J.-L. (2024). Definitions of Chronic Disease Need to Be More Patient Centred. BMJ.

[2] Fan Y., Pedersen O. (2021). Gut Microbiota in Human Metabolic Health and Disease. Nat. Rev. Microbiol..

[3] Wu J., Wang K., Wang X., Pang Y., Jiang C. (2021). The Role of the Gut Microbiome and Its Metabolites in Metabolic Diseases. Protein Cell.

[4] Xie Q. (2022). Effect of Coptis Chinensis Franch and Magnolia Officinalis on Intestinal Flora and Intestinal Barrier in a TNBS-Induced Ulcerative Colitis Rats Model. Phytomedicine.

[5] Liu Y., Zhou M., Yang M., Jin C., Song Y., Chen J., Gao M., Ai Z., Su D. (2021). Pulsatilla Chinensis Saponins Ameliorate Inflammation and DSS-Induced Ulcerative Colitis in Rats by Regulating the Composition and Diversity of Intestinal Flora. Front. Cell. Infect. Microbiol..

[6] Chen Y. (2021). Role and Mechanism of Gut Microbiota in Human Disease. Front. Cell Infect. Microbiol..

[7] Agus A., Clément K., Sokol H. (2021). Gut Microbiota-Derived Metabolites as Central Regulators in Metabolic Disorders. Gut.

[8] Jiang S. (2021). Gut Microbiota Dependent Trimethylamine N-Oxide Aggravates Angiotensin II–Induced Hypertension. Redox Biol..

[9] Zou Y., Song X., Liu N., Sun W., Liu B. (2022). Intestinal Flora: A Potential New Regulator of Cardiovascular Disease. Aging Dis..

[10] Li X.S., Obeid S., Klingenberg R., Gencer B., Mach F., Räber L., Windecker S., Rodondi N., Nanchen D., Muller O. (2017). Gut Microbiota-Dependent Trimethylamine N-Oxide in Acute Coronary Syndromes: A Prognostic Marker for Incident Cardiovascular Events beyond Traditional Risk Factors. Eur. Heart J..

[11] Bi S.-H., Su C., Yang P., Zhang X., Wang Y., Tang W., Yang W., He L. (2023). Higher Serum Trimethylamine N-Oxide (TMAO) Levels Are Associated with Increased Visceral Fat in Hemodialysis Patients. CN.

[12] Kong L., Zhao Q., Jiang X., Hu J., Jiang Q., Sheng L., Peng X., Wang S., Chen Y., Wan Y. (2024). Trimethylamine N-Oxide Impairs β-Cell Function and Glucose Tolerance. Nat. Commun..

[13] Nian F., Chen Y., Xia Q., Zhu C., Wu L., Lu X. (2024). Gut Microbiota Metabolite Trimethylamine N-Oxide Promoted NAFLD Progression by Exacerbating Intestinal Barrier Disruption and Intrahepatic Cellular Imbalance. Int. Immunopharmacol..

[14] Yue C., Yang X., Li J., Chen X., Zhao X., Chen Y., Wen Y. (2017). Trimethylamine N-Oxide Prime NLRP3 Inflammasome via Inhibiting ATG16L1-Induced Autophagy in Colonic Epithelial Cells. Biochem. Biophys. Res. Commun..

[15] Hsu C.-N., Yu H.-R., Lin I.-C., Tiao M.-M., Huang L.-T., Hou C.-Y., Chang-Chien G.-P., Lin S., Tain Y.-L. (2022). Sodium Butyrate Modulates Blood Pressure and Gut Microbiota in Maternal Tryptophan-Free Diet-Induced Hypertension Rat Offspring. J. Nutr. Biochem..

[16] Wang Y., Dou W., Qian X., Chen H., Zhang Y., Yang L., Wu Y., Xu X. (2025). Advancements in the Study of Short-Chain Fatty Acids and Their Therapeutic Effects on Atherosclerosis. Life Sci..

[17] Furukawa N., Kobayashi M., Ito M., Matsui H., Ohashi K., Murohara T., Takeda J., Ueyama J., Hirayama M., Ohno K. (2024). Soy Protein β-Conglycinin Ameliorates Pressure Overload-Induced Heart Failure by Increasing Short-Chain Fatty Acid (SCFA)-Producing Gut Microbiota and Intestinal SCFAs. Clin. Nutr..

[18] Su C.-W., Chen C.-Y., Mao T., Chen N., Steudel N., Jiao L., Lan J., Fasano A., Walker W.A., Shi H.N. (2023). Maternal Helminth Infection Protects Offspring from High-Fat-Diet-Induced Obesity through Altered Microbiota and SCFAs. Cell Mol. Immunol..

[19] Zhao L., Zhang F., Ding X., Wu G., Lam Y.Y., Wang X., Fu H., Xue X., Lu C., Ma J. (2018). Gut Bacteria Selectively Promoted by Dietary Fibers Alleviate Type 2 Diabetes. Science.

[20] Hong Y., Sheng L., Zhong J., Tao X., Zhu W., Ma J., Yan J., Zhao A., Zheng X., Wu G. (2021). Desulfovibrio Vulgaris, a Potent Acetic Acid-Producing Bacterium, Attenuates Nonalcoholic Fatty Liver Disease in Mice. Gut Microbes.

[21] Parada Venegas D., De La Fuente M.K., Landskron G., González M.J., Quera R., Dijkstra G., Harmsen H.J.M., Faber K.N., Hermoso M.A. (2019). Short Chain Fatty Acids (SCFAs)-Mediated Gut Epithelial and Immune Regulation and Its Relevance for Inflammatory Bowel Diseases. Front. Immunol..

[22] Li Y., Gao Y.-N., Zhu Y.-B., Lu W.-F., Yu J.-Y., Dong Y.-Y., Xu M.-Y., Peng B., Wu J.-Z., Su Q. (2024). Taurocholic Acid Ameliorates Hypertension through the Activation of TGR5 in the Hypothalamic Paraventricular Nucleus. Food Funct..

[23] Zhou X., Zhou X., Zhang Z., Zhu R., Lu M., Lv K., Fang C., Ming Z., Cheng Z., Hu Y. (2024). Mechanism of Bile Acid in Regulating Platelet Function and Thrombotic Diseases. Adv. Sci..

[24] Von Haehling S., Schefold J.C., Jankowska E.A., Springer J., Vazir A., Kalra P.R., Sandek A., Fauler G., Stojakovic T., Trauner M. (2012). Ursodeoxycholic acid in patients with chronic heart failure. a double-blind, randomized, placebo-controlled, crossover trial. J. Am. Coll. Cardiol..

[25] Li X., Yang J., Zhou X., Dai C., Kong M., Xie L., Liu C., Liu Y., Li D., Ma X. (2024). Ketogenic Diet-Induced Bile Acids Protect against Obesity through Reduced Calorie Absorption. Nat. Metab..

[26] Liu T., Zhao M., Zhang Y., Xu R., Fu Z., Jin T., Song J., Huang Y., Wang M., Zhao C. (2024). Polysaccharides from Phellinus Linteus Attenuate Type 2 Diabetes Mellitus in Rats via Modulation of Gut Microbiota and Bile Acid Metabolism. Int. J. Biol. Macromol..

[27] Gillard J., Clerbaux L.-A., Nachit M., Sempoux C., Staels B., Bindels L.B., Tailleux A., Leclercq I.A. (2022). Bile Acids Contribute to the Development of Non-Alcoholic Steatohepatitis in Mice. JHEP Rep..

[28] Dong S., Zhu M., Wang K., Zhao X., Hu L., Jing W., Lu H., Wang S. (2021). Dihydromyricetin Improves DSS-Induced Colitis in Mice via Modulation of Fecal-Bacteria-Related Bile Acid Metabolism. Pharmacol. Res..

[29] Qin J., Li R., Raes J., Arumugam M., Burgdorf K.S., Manichanh C., Nielsen T., Pons N., Levenez F., Yamada T. (2010). A Human Gut Microbial Gene Catalogue Established by Metagenomic Sequencing. Nature.

[30] Human Microbiome Project Consortium (2012). Structure, Function and Diversity of the Healthy Human Microbiome. Nature.

[31] Eiseman B., Silen W., Bascom G.S., Kauvar A.J. (1958). Fecal Enema as an Adjunct in the Treatment of Pseudomembranous Enterocolitis. Surgery..

[32] Schaedler R.W., Costello R. (1965). Association of germfree mice with bacteria isolated from normal mice. J. Exp. Med..

[33] De Vos W.M., Tilg H., Van Hul M., Cani P.D. (2022). Gut Microbiome and Health: Mechanistic Insights. Gut.

[34] de Groot P., Nikolic T., Pellegrini S., Sordi V., Imangaliyev S., Rampanelli E., Hanssen N., Attaye I., Bakker G., Duinkerken G. (2021). Faecal microbiota transplantation halts progression of human new-onset type 1 diabetes in a randomised controlled trial. Gut.

[35] Cotillard A., Kennedy S.P., Kong L.C., Prifti E., Pons N., Le Chatelier E., Almeida M., Quinquis B., Levenez F., Galleron N. (2013). Dietary Intervention Impact on Gut Microbial Gene Richness. Nature.

[36] Turnbaugh P.J., Ley R.E., Mahowald M.A., Magrini V., Mardis E.R., Gordon J.I. (2006). An Obesity-Associated Gut Microbiome with Increased Capacity for Energy Harvest. Nature.

[37] Aron-Wisnewsky J., Warmbrunn M.V., Nieuwdorp M., Clément K. (2021). Metabolism and Metabolic Disorders and the Microbiome: The Intestinal Microbiota Associated With Obesity, Lipid Metabolism, and Metabolic Health—Pathophysiology and Therapeutic Strategies. Gastroenterology.

[38] Wang Y. (2023). Fecal Microbiota Transplantation Attenuates Escherichia Coli Infected Outgrowth by Modulating the Intestinal Microbiome. Microb. Cell Factories.

[39] Liu Y., Dai M. (2020). Trimethylamine N-Oxide Generated by the Gut Microbiota Is Associated with Vascular Inflammation: New Insights into Atherosclerosis. Mediat. Inflamm..

[40] Tomlinson J.A.P., Wheeler D.C. (2017). The Role of Trimethylamine N-Oxide as a Mediator of Cardiovascular Complications in Chronic Kidney Disease. Kidney Int..

[41] Akhtar M., Chen Y., Ma Z., Zhang X., Shi D., Khan J.A., Liu H. (2022). Gut Microbiota-Derived Short Chain Fatty Acids Are Potential Mediators in Gut Inflammation. Anim. Nutr..

[42] He J., Zhang P., Shen L., Niu L., Tan Y., Chen L., Zhao Y., Bai L., Hao X., Li X. (2020). Short-Chain Fatty Acids and Their Association with Signalling Pathways in Inflammation, Glucose and Lipid Metabolism. Int. J. Mol. Sci..

[43] Koh A., De Vadder F., Kovatcheva-Datchary P., Bäckhed F. (2016). From Dietary Fiber to Host Physiology: Short-Chain Fatty Acids as Key Bacterial Metabolites. Cell.

[44] Seethaler B., Nguyen N.K., Basrai M., Kiechle M., Walter J., Delzenne N.M., Bischoff S.C. (2022). Short-Chain Fatty Acids Are Key Mediators of the Favorable Effects of the Mediterranean Diet on Intestinal Barrier Integrity: Data from the Randomized Controlled LIBRE Trial. Am. J. Clin. Nutr..

[45] Shapiro H., Kolodziejczyk A.A., Halstuch D., Elinav E. (2018). Bile Acids in Glucose Metabolism in Health and Disease. J. Exp. Med..

[46] Chávez-Talavera O., Tailleux A., Lefebvre P., Staels B. (2017). Bile Acid Control of Metabolism and Inflammation in Obesity, Type 2 Diabetes, Dyslipidemia, and Nonalcoholic Fatty Liver Disease. Gastroenterology.

[47] Jia W., Xie G., Jia W. (2018). Bile Acid–Microbiota Crosstalk in Gastrointestinal Inflammation and Carcinogenesis. Nat. Rev. Gastroenterol. Hepatol..

[48] Collins S.L., Stine J.G., Bisanz J.E., Okafor C.D., Patterson A.D. (2023). Bile Acids and the Gut Microbiota: Metabolic Interactions and Impacts on Disease. Nat. Rev. Microbiol..

[49] Nemet I. (2020). A Cardiovascular Disease-Linked Gut Microbial Metabolite Acts via Adrenergic Receptors. Cell.

[50] Zhu Y., Dwidar M., Nemet I., Buffa J.A., Sangwan N., Li X.S., Anderson J.T., Romano K.A., Fu X., Funabashi M. (2023). Two Distinct Gut Microbial Pathways Contribute to Meta-Organismal Production of Phenylacetylglutamine with Links to Cardiovascular Disease. Cell Host Microbe.

[51] Vanweert F., Schrauwen P., Phielix E. (2022). Role of Branched-Chain Amino Acid Metabolism in the Pathogenesis of Obesity and Type 2 Diabetes-Related Metabolic Disturbances BCAA Metabolism in Type 2 Diabetes. Nutr. Diabetes.

[52] Nie C., He T., Zhang W., Zhang G., Ma X. (2018). Branched Chain Amino Acids: Beyond Nutrition Metabolism. Int. J. Mol. Sci..

[53] Holeček M. (2018). Branched-Chain Amino Acids in Health and Disease: Metabolism, Alterations in Blood Plasma, and as Supplements. Nutr. Metab..

[54] Sadok I., Jędruchniewicz K. (2023). Dietary Kynurenine Pathway Metabolites—Source, Fate, and Chromatographic Determinations. Int. J. Mol. Sci..

[55] Ala M. (2022). Tryptophan Metabolites Modulate Inflammatory Bowel Disease and Colorectal Cancer by Affecting Immune System. Int. Rev. Immunol..

[56] Joisten N., Ruas J.L., Braidy N., Guillemin G.J., Zimmer P. (2021). The Kynurenine Pathway in Chronic Diseases: A Compensatory Mechanism or a Driving Force?. Trends Mol. Med..

[57] Pathak S., Nadar R., Kim S., Liu K., Govindarajulu M., Cook P., Alexander C.S.W., Dhanasekaran M., Moore T. (2024). The Influence of Kynurenine Metabolites on Neurodegenerative Pathologies. Int. J. Biol. Macromol..

[58] Li M., Ding Y., Wei J., Dong Y., Wang J., Dai X., Yan J., Chu F., Zhang K., Meng F. (2024). Gut Microbiota Metabolite Indole-3-Acetic Acid Maintains Intestinal Epithelial Homeostasis through Mucin Sulfation. Gut Microbes.

[59] Ji Y., Gao Y., Chen H., Yin Y., Zhang W. (2019). Indole-3-Acetic Acid Alleviates Nonalcoholic Fatty Liver Disease in Mice via Attenuation of Hepatic Lipogenesis, and Oxidative and Inflammatory Stress. Nutrients.

[60] Murray C.J.L. (2022). The Global Burden of Disease Study at 30 Years. Nat. Med..

[61] Mazur M. (2024). Dietary Strategies for Cardiovascular Disease Risk Factors Prevention. Curr. Probl. Cardiol..

[62] Tsao C.W., Aday A.W., Almarzooq Z.I., Alonso A., Beaton A.Z., Bittencourt M.S., Boehme A.K., Buxton A.E., Carson A.P., Commodore-Mensah Y. (2022). Heart Disease and Stroke Statistics—2022 Update: A Report From the American Heart Association. Circulation.

[63] Rout A., Duhan S., Umer M., Li M., Kalra D. (2024). Atherosclerotic Cardiovascular Disease Risk Prediction: Current State-of-the-Art. Heart.

[64] Timmis A., Group C.W. (2022). European Society of Cardiology: Cardiovascular Disease Statistics 2021. Eur. Heart J..

[65] Witkowski M., Weeks T.L., Hazen S.L. (2020). Gut Microbiota and Cardiovascular Disease. Circ. Res..

[66] Bs M.C., Mm L.L., Bs S.L., Chen L., Bs F.J., Mm Y.P., Lin Y. (2023). Gut Microbiota Changes in Patients with Hypertension: A Systematic Review and Meta-Analysis. J. Clin. Hypertens..

[67] Li J. (2017). Gut Microbiota Dysbiosis Contributes to the Development of Hypertension. Microbiome.

[68] Santisteban M.M., Qi Y., Zubcevic J., Kim S., Yang T., Shenoy V., Cole-Jeffrey C.T., Lobaton G.O., Stewart D.C., Rubiano A. (2017). Hypertension-Linked Pathophysiological Alterations in the Gut. Circ. Res..

[69] Li Y., Zhou E., Yu Y., Wang B., Zhang L., Lei R., Xue B., Tian X., Niu J., Liu J. (2024). Butyrate Attenuates Cold-Induced Hypertension via Gut Microbiota and Activation of Brown Adipose Tissue. Sci. Total Environ..

[70] Bardhan P., Mei X., Lai N.K., Mell B., Tummala R., Aryal S., Manandhar I., Hwang H., Jhuma T.A., Atluri R.R. (2024). Salt Responsive Gut Microbiota Induces Sex Specific Blood Pressure Changes. Circ. Res..

[71] Jie Z., Xia H., Zhong S.-L., Feng Q., Li S., Liang S., Zhong H., Liu Z., Gao Y., Zhao H. (2017). The Gut Microbiome in Atherosclerotic Cardiovascular Disease. Nat. Commun..

[72] Zhao S., Zhou L., Wang Q., Cao J.-H., Chen Y., Wang W., Zhu B.-D., Wei Z.-H., Li R., Li C.-Y. (2023). Elevated Branched-Chain Amino Acid Promotes Atherosclerosis Progression by Enhancing Mitochondrial-to-Nuclear H2O2-Disulfide HMGB1 in Macrophages. Redox Biol..

[73] Qiao S., Liu C., Sun L., Wang T., Dai H., Wang K., Bao L., Li H., Wang W., Liu S.-J. (2022). Gut Parabacteroides Merdae Protects against Cardiovascular Damage by Enhancing Branched-Chain Amino Acid Catabolism. Nat. Metab..

[74] Canyelles M., Borràs C., Rotllan N., Tondo M., Escolà-Gil J.C., Blanco-Vaca F. (2023). Gut Microbiota-Derived TMAO: A Causal Factor Promoting Atherosclerotic Cardiovascular Disease?. Int. J. Mol. Sci..

[75] Wang Z. (2011). Gut Flora Metabolism of Phosphatidylcholine Promotes Cardiovascular Disease. Nature.

[76] Zhu W., Gregory J.C., Org E., Buffa J.A., Gupta N., Wang Z., Li L., Fu X., Wu Y., Mehrabian M. (2016). Gut Microbial Metabolite TMAO Enhances Platelet Hyperreactivity and Thrombosis Risk. Cell.

[77] Seldin M.M., Meng Y., Qi H., Zhu W., Wang Z., Hazen S.L., Lusis A.J., Shih D.M. (2016). Trimethylamine N--Oxide Promotes Vascular Inflammation Through Signaling of Mitogen--Activated Protein Kinase and Nuclear Factor--κB. JAHA.

[78] Bertero E., Maack C. (2018). Metabolic remodelling in heart failure. Nat. Rev. Cardiol..

[79] Dai H., Hou T., Wang Q., Hou Y., Wang T., Zheng J., Lin H., Zhao Z., Li M., Wang S. (2023). Causal Relationships between the Gut Microbiome, Blood Lipids, and Heart Failure: A Mendelian Randomization Analysis. Eur. J. Prev. Cardiol..

[80] Cui X., Ye L., Li J., Jin L., Wang W., Li S., Bao M., Wu S., Li L., Geng B. (2018). Metagenomic and Metabolomic Analyses Unveil Dysbiosis of Gut Microbiota in Chronic Heart Failure Patients. Sci. Rep..

[81] Romano K.A., Nemet I., Prasad Saha P., Haghikia A., Li X.S., Mohan M.L., Lovano B., Castel L., Witkowski M., Buffa J.A. (2023). Gut Microbiota-Generated Phenylacetylglutamine and Heart Failure. Circ. Heart Fail..

[82] Ilardi F., Gargiulo G., Schiattarella G.G., Giugliano G., Paolillo R., Menafra G., De Angelis E., Scudiero L., Franzone A., Stabile E. (2018). Effects of Carvedilol Versus Metoprolol on Platelet Aggregation in Patients With Acute Coronary Syndrome: The PLATE-BLOCK Study. Am. J. Cardiol..

[83] Marques F.Z., Nelson E., Chu P.-Y., Horlock D., Fiedler A., Ziemann M., Tan J.K., Kuruppu S., Rajapakse N.W., El-Osta A. (2017). High-Fiber Diet and Acetate Supplementation Change the Gut Microbiota and Prevent the Development of Hypertension and Heart Failure in Hypertensive Mice. Circulation.

[84] Jarmukhanov Z., Mukhanbetzhanov N., Kozhakhmetov S., Nurgaziyev M., Sailybayeva A., Bekbossynova M., Kushugulova A. (2024). The Association between the Gut Microbiota Metabolite Trimethylamine N-Oxide and Heart Failure. Front. Microbiol..

[85] Clemente-Suárez V.J., Martín-Rodríguez A., Redondo-Flórez L., López-Mora C., Yáñez-Sepúlveda R., Tornero-Aguilera J.F. (2023). New Insights and Potential Therapeutic Interventions in Metabolic Diseases. Int. J. Mol. Sci..

[86] Li D., Li Y., Yang S., Lu J., Jin X., Wu M. (2022). Diet-gut microbiota-epigenetics in metabolic diseases: From mechanisms to therapeutics. Biomed Pharmacother..

[87] Ballini A., Scacco S., Boccellino M., Santacroce L., Arrigoni R. (2020). Microbiota and Obesity: Where Are We Now?. Biology.

[88] Ke W., Flay K.J., Huang X., Hu X., Chen F., Li C., Yang D.A. (2023). Polysaccharides from Platycodon Grandiflorus Attenuates High-Fat Diet Induced Obesity in Mice through Targeting Gut Microbiota. Biomed. Pharmacother..

[89] Zhu M., Ouyang J., Zhou F., Zhao C., Zhu W., Liu C., Huang P., Li J., Tang J., Zhang Z. (2023). Polysaccharides from Fu Brick Tea Ameliorate Obesity by Modulating Gut Microbiota and Gut Microbiota-Related Short Chain Fatty Acid and Amino Acid Metabolism. J. Nutr. Biochem..

[90] Zhang X.-Y., Chen J., Yi K., Peng L., Xie J., Gou X., Peng T., Tang L. (2020). Phlorizin Ameliorates Obesity-Associated Endotoxemia and Insulin Resistance in High-Fat Diet-Fed Mice by Targeting the Gut Microbiota and Intestinal Barrier Integrity. Gut Microbes.

[91] Ma L., Ni Y., Wang Z., Tu W., Ni L., Zhuge F., Zheng A., Hu L., Zhao Y., Zheng L. (2020). Spermidine Improves Gut Barrier Integrity and Gut Microbiota Function in Diet-Induced Obese Mice. Gut Microbes.

[92] Pluznick J.L. (2016). Gut Microbiota in Renal Physiology: Focus on Short-Chain Fatty Acids and Their Receptors. Kidney Int..

[93] Nakajima A., Nakatani A., Hasegawa S., Irie J., Ozawa K., Tsujimoto G., Suganami T., Itoh H., Kimura I. (2017). The Short Chain Fatty Acid Receptor GPR43 Regulates Inflammatory Signals in Adipose Tissue M2-Type Macrophages. PLoS ONE.

[94] Kimura I., Ozawa K., Inoue D., Imamura T., Kimura K., Maeda T., Terasawa K., Kashihara D., Hirano K., Tani T. (2013). The Gut Microbiota Suppresses Insulin-Mediated Fat Accumulation via the Short-Chain Fatty Acid Receptor GPR43. Nat. Commun..

[95] He Z., Guo J., Zhang H., Yu J., Zhou Y., Wang Y., Li T., Yan M., Li B., Chen Y. (2023). Atractylodes macrocephala Koidz polysaccharide improves glycolipid metabolism disorders through activation of aryl hydrocarbon receptor by gut flora-produced tryptophan metabolites. Int. J. Biol. Macromol..

[96] Zhao H., Li M., Liu L., Li D., Zhao L., Wu Z., Zhou M., Jia L., Yang F. (2023). Cordyceps Militaris Polysaccharide Alleviates Diabetic Symptoms by Regulating Gut Microbiota against TLR4/NF-κB Pathway. Int. J. Biol. Macromol..

[97] Zhou W., Yang T., Xu W., Huang Y., Ran L., Yan Y., Mi J., Lu L., Sun Y., Zeng X. (2022). The polysaccharides from the fruits of *Lycium barbarum* L. confer anti-diabetic effect by regulating gut microbiota and intestinal barrier. Carbohydr. Polym..

[98] Baars D.P., Fondevila M.F., Meijnikman A.S., Nieuwdorp M. (2024). The Central Role of the Gut Microbiota in the Pathophysiology and Management of Type 2 Diabetes. Cell Host Microbe.

[99] Mandaliya D.K., Seshadri S. (2019). Short Chain Fatty Acids, Pancreatic Dysfunction and Type 2 Diabetes. Pancreatology.

[100] Bauer K.C., Littlejohn P.T., Ayala V., Creus-Cuadros A., Finlay B.B. (2022). Nonalcoholic Fatty Liver Disease and the Gut-Liver Axis: Exploring an Undernutrition Perspective. Gastroenterology.

[101] Eslam M., Newsome P.N., Sarin S.K., Anstee Q.M., Targher G., Romero-Gomez M., Zelber-Sagi S., Wai-Sun Wong V., Dufour J.-F., Schattenberg J.M. (2020). A New Definition for Metabolic Dysfunction-Associated Fatty Liver Disease: An International Expert Consensus Statement. J. Hepatol..

[102] Eslam M., Sanyal A.J., George J., Sanyal A., Neuschwander-Tetri B., Tiribelli C., Kleiner D.E., Brunt E., Bugianesi E., Yki-Järvinen H. (2020). MAFLD: A Consensus-Driven Proposed Nomenclature for Metabolic Associated Fatty Liver Disease. Gastroenterology.

[103] Zhang Y., Wang X., Lin J., Liu J., Wang K., Nie Q., Ye C., Sun L., Ma Y., Qu R. (2024). A Microbial Metabolite Inhibits the HIF-2α-Ceramide Pathway to Mediate the Beneficial Effects of Time-Restricted Feeding on MASH. Cell Metab..

[104] Sun B., Jia Y., Hong J., Sun Q., Gao S., Hu Y., Zhao N., Zhao R. (2018). Sodium Butyrate Ameliorates High-Fat-Diet-Induced Non-Alcoholic Fatty Liver Disease through Peroxisome Proliferator-Activated Receptor α-Mediated Activation of β Oxidation and Suppression of Inflammation. J. Agric. Food Chem..

[105] Liu W., Luo X., Tang J., Mo Q., Zhong H., Zhang H., Feng F. (2021). A Bridge for Short-Chain Fatty Acids to Affect Inflammatory Bowel Disease, Type 1 Diabetes, and Non-Alcoholic Fatty Liver Disease Positively: By Changing Gut Barrier. Eur. J. Nutr..

[106] Min B.H., Devi S., Kwon G.H., Gupta H., Jeong J.-J., Sharma S.P., Won S.-M., Oh K.-K., Yoon S.J., Park H.J. (2024). Gut Microbiota-Derived Indole Compounds Attenuate Metabolic Dysfunction-Associated Steatotic Liver Disease by Improving Fat Metabolism and Inflammation. Gut Microbes.

[107] Federici S., Kviatcovsky D., Valdés-Mas R., Elinav E. (2023). Microbiome-Phage Interactions in Inflammatory Bowel Disease. Clin. Microbiol. Infect..

[108] Bretto E., Ribaldone D.G., Caviglia G.P., Saracco G.M., Bugianesi E., Frara S. (2023). Inflammatory Bowel Disease: Emerging Therapies and Future Treatment Strategies. Biomedicines.

[109] Lloyd-Price J., Arze C., Ananthakrishnan A.N., Schirmer M., Avila-Pacheco J., Poon T.W., Andrews E., Ajami N.J., Bonham K.S., IBDMDB Investigators (2019). Multi-Omics of the Gut Microbial Ecosystem in Inflammatory Bowel Diseases. Nature.

[110] Franzosa E.A., Sirota-Madi A., Avila-Pacheco J., Fornelos N., Haiser H.J., Reinker S., Vatanen T., Hall A.B., Mallick H., McIver L.J. (2018). Gut Microbiome Structure and Metabolic Activity in Inflammatory Bowel Disease. Nat. Microbiol..

[111] Yang M., Gu Y., Li L., Liu T., Song X., Sun Y., Cao X., Wang B., Jiang K., Cao H. (2021). Bile Acid–Gut Microbiota Axis in Inflammatory Bowel Disease: From Bench to Bedside. Nutrients.

[112] Yuan M., Chang L., Gao P., Li J., Lu X., Hua M., Li X., Liu X., Lan Y. (2024). Synbiotics containing sea buckthorn polysaccharides ameliorate DSS-induced colitis in mice via regulating Th17/Treg homeostasis through intestinal microbiota and their production of BA metabolites and SCFAs. Int. J. Biol. Macromol..

[113] Zheng M., Zhai Y., Yu Y., Shen J., Chu S., Focaccia E., Tian W., Wang S., Liu X., Yuan X. (2024). TNF Compromises Intestinal Bile-Acid Tolerance Dictating Colitis Progression and Limited Infliximab Response. Cell Metab..

[114] Ozturk O., Celebi G., Duman U.G., Kupcuk E., Uyanik M., Sertoglu E. (2024). Short-Chain Fatty Acid Levels in Stools of Patients with Inflammatory Bowel Disease Are Lower than Those in Healthy Subjects. Eur. J. Gastroenterol. Hepatol..

[115] Li G., Lin J., Zhang C., Gao H., Lu H., Gao X., Zhu R., Li Z., Li M., Liu Z. (2021). Microbiota Metabolite Butyrate Constrains Neutrophil Functions and Ameliorates Mucosal Inflammation in Inflammatory Bowel Disease. Gut Microbes.

[116] Chen G., Ran X., Li B., Li Y., He D., Huang B., Fu S., Liu J., Wang W. (2018). Sodium Butyrate Inhibits Inflammation and Maintains Epithelium Barrier Integrity in a TNBS-Induced Inflammatory Bowel Disease Mice Model. eBioMedicine.

[117] Dupraz L., Magniez A., Rolhion N., Richard M.L., Da Costa G., Touch S., Mayeur C., Planchais J., Agus A., Danne C. (2021). Gut Microbiota-Derived Short-Chain Fatty Acids Regulate IL-17 Production by Mouse and Human Intestinal Γδ T Cells. Cell Rep..

[118] Wang S., Van Schooten F.-J., Jin H., Jonkers D., Godschalk R. (2023). The Involvement of Intestinal Tryptophan Metabolism in Inflammatory Bowel Disease Identified by a Meta-Analysis of the Transcriptome and a Systematic Review of the Metabolome. Nutrients.

[119] Pernomian L., Duarte-Silva M., De Barros Cardoso C.R. (2020). The Aryl Hydrocarbon Receptor (AHR) as a Potential Target for the Control of Intestinal Inflammation: Insights from an Immune and Bacteria Sensor Receptor. Clin. Rev. Allerg. Immunol..

[120] Xie L.-W., Cai S., Lu H.-Y., Tang F.-L., Zhu R.-Q., Tian Y., Li M. (2024). Microbiota-Derived I3A Protects the Intestine against Radiation Injury by Activating AhR/IL-10/Wnt Signaling and Enhancing the Abundance of Probiotics. Gut Microbes.

[121] Hu X., Xiao W., Lei Y., Green A., Lee X., Maradana M.R., Gao Y., Xie X., Wang R., Chennell G. (2023). Aryl Hydrocarbon Receptor Utilises Cellular Zinc Signals to Maintain the Gut Epithelial Barrier. Nat. Commun..

[122] Gao Y., Liu K.-Y., Xiao W., Xie X., Liang Q., Tu Z., Yang L., Yu H., Guo H., Huang S. (2024). Aryl Hydrocarbon Receptor Confers Protection against Macrophage Pyroptosis and Intestinal Inflammation through Regulating Polyamine Biosynthesis. Theranostics.

[123] Wang G., Fan Y., Zhang G., Cai S., Ma Y., Yang L., Wang Y., Yu H., Qiao S., Zeng X. (2024). Microbiota-Derived Indoles Alleviate Intestinal Inflammation and Modulate Microbiome by Microbial Cross-Feeding. Microbiome.

[124] Jiang L., Hao Y., Han D., Dong W., Yang A., Sun Z., Ge Y., Duan S., Zhang X., Dai Z. (2024). Gut Microbiota Dysbiosis Deteriorates Immunoregulatory Effects of Tryptophan via Colonic Indole and LBP/HTR2B-Mediated Macrophage Function. ISME J..

[125] Feng R., Tian Z., Mao R., Ma R., Luo W., Zhao M., Li X., Liu Y., Huang K., Xiang L. (2023). Gut Microbiome-Generated Phenylacetylglutamine from Dietary Protein is Associated with Crohn’s Disease and Exacerbates Colitis in Mouse Model Possibly via Platelet Activation. J. Crohn’s Colitis.

[126] Wang X., Lin S., Wang L., Cao Z., Zhang M., Zhang Y., Liu R., Liu J. (2023). Versatility of Bacterial Outer Membrane Vesicles in Regulating Intestinal Homeostasis. Sci. Adv..

[127] Taladrid D., de Celis M., Belda I., Bartolomé B., Moreno-Arribas M.V. (2022). Hypertension- and Glycaemia-Lowering Effects of a Grape-Pomace-Derived Seasoning in High- Cardiovascular Risk and Healthy Subjects. Interplay with the Gut Microbiome. Food Funct..

[128] Gomez-Arango L.F., Barrett H.L., McIntyre H.D., Callaway L.K., Morrison M., Nitert M.D. (2016). Increased Systolic and Diastolic Blood Pressure Is Associated With Altered Gut Microbiota Composition and Butyrate Production in Early Pregnancy. Hypertension.

[129] Dan X., Mushi Z., Baili W., Han L., Enqi W., Huanhu Z., Shuchun L. (2019). Differential Analysis of Hypertension-Associated Intestinal Microbiota. Int. J. Med. Sci..

[130] Nash D.B. (2023). The Future of Chronic Disease Management. Popul. Health Manag..

[131] Dale M.T., Elkins M.R. (2021). Chronic Disease. J. Physiother..

[132] Luedde M., Winkler T., Heinsen F.A., Rühlemann M.C., Spehlmann M.E., Bajrovic A., Lieb W., Franke A., Ott S.J., Frey N. (2017). Heart Failure Is Associated with Depletion of Core Intestinal Microbiota. ESC Heart Fail..

[133] Hibberd A.A., Yde C.C., Ziegler M.L., Honoré A.H., Saarinen M.T., Lahtinen S., Stahl B., Jensen H.M., Stenman L.K. (2019). Probiotic or Synbiotic Alters the Gut Microbiota and Metabolism in a Randomised Controlled Trial of Weight Management in Overweight Adults. BM.

[134] Crovesy L., El-Bacha T., Rosado E.L. (2021). Modulation of the Gut Microbiota by Probiotics and Symbiotics Is Associated with Changes in Serum Metabolite Profile Related to a Decrease in Inflammation and Overall Benefits to Metabolic Health: A Double-Blind Randomized Controlled Clinical Trial in Women with Obesity. Food Funct..

[135] Lee Y., Cho J.-Y., Cho K.Y. (2023). Serum, Urine, and Fecal Metabolome Alterations in the Gut Microbiota in Response to Lifestyle Interventions in Pediatric Obesity: A Non-Randomized Clinical Trial. Nutrients.

[136] Liu G., Liang L., Yu G., Li Q. (2018). Pumpkin Polysaccharide Modifies the Gut Microbiota during Alleviation of Type 2 Diabetes in Rats. Int. J. Biol. Macromol..

[137] Xia T., Liu C.-S., Hu Y.-N., Luo Z.-Y., Chen F.-L., Yuan L.-X., Tan X.-M. (2021). Coix Seed Polysaccharides Alleviate Type 2 Diabetes Mellitus via Gut Microbiota-Derived Short-Chain Fatty Acids Activation of IGF1/PI3K/AKT Signaling. Food Res. Int..

[138] Luo L., Zhang H., Chen W., Zheng Z., He Z., Wang H., Wang K., Zhang Y. (2023). Angelica sinensis Polysaccharide Ameliorates Nonalcoholic Fatty Liver Disease via Restoring Estrogen-Related Receptor α Expression in Liver. Phytother. Res..

[139] Wang X., Shi L., Wang X., Feng Y., Wang Y. (2019). MDG-1, an Ophiopogon Polysaccharide, Restrains Process of Non-Alcoholic Fatty Liver Disease via Modulating the Gut-Liver Axis. Int. J. Biol. Macromol..

[140] He C., Wang H., Liao W.-D., Peng C., Shu X., Zhu X., Zhu Z.-H. (2019). Characteristics of Mucosa-Associated Gut Microbiota during Treatment in Crohn’s Disease. WJG.

[141] Wu J., Wei Z., Cheng P., Qian C., Xu F., Yang Y., Wang A., Chen W., Sun Z., Lu Y. (2020). Rhein Modulates Host Purine Metabolism in Intestine through Gut Microbiota and Ameliorates Experimental Colitis. Theranostics.

[142] Henn M.R., O’Brien E.J., Diao L., Feagan B.G., Sandborn W.J., Huttenhower C., Wortman J.R., McGovern B.H., Wang-Weigand S., Lichter D.I. (2021). A Phase 1b Safety Study of SER-287, a Spore-Based Microbiome Therapeutic, for Active Mild to Moderate Ulcerative Colitis. Gastroenterology.

[143] Yuan X., Chen B., Duan Z., Xia Z., Ding Y., Chen T., Liu H., Wang B., Yang B., Wang X. (2021). Depression and Anxiety in Patients with Active Ulcerative Colitis: Crosstalk of Gut Microbiota, Metabolomics and Proteomics. Gut Microbes.

[144] Ross F.C., Patangia D., Grimaud G., Lavelle A., Dempsey E.M., Ross R.P., Stanton C. (2024). The interplay between diet and the gut microbiome: Implications for health and disease. Nat. Rev. Microbiol..

[145] Ghosh T.S., Rampelli S., Jeffery I.B., Santoro A., Neto M., Capri M., Giampieri E., Jennings A., Candela M., Turroni S. (2020). Mediterranean Diet Intervention Alters the Gut Microbiome in Older People Reducing Frailty and Improving Health Status: The NU-AGE 1-Year Dietary Intervention across Five European Countries. Gut.

[146] Rondanelli M., Gasparri C., Peroni G., Faliva M.A., Naso M., Perna S., Bazire P., Sajuox I., Maugeri R., Rigon C. (2021). The Potential Roles of Very Low Calorie, Very Low Calorie Ketogenic Diets and Very Low Carbohydrate Diets on the Gut Microbiota Composition. Front. Endocrinol..

[147] Bock P.M., Martins A.F., Schaan B.D. (2024). Understanding How Pre- and Probiotics Affect the Gut Microbiome and Metabolic Health. Am. J. Physiol.-Endocrinol. Metab..

[148] Moszak M., Szulińska M., Bogdański P. (2020). You Are What You Eat—The Relationship between Diet, Microbiota, and Metabolic Disorders—A Review. Nutrients.

[149] Haque M., Kaminsky L., Abdulqadir R., Engers J., Kovtunov E., Rawat M., Al-Sadi R., Ma T.Y. (2024). *Lactobacillus acidophilus* inhibits the TNF-α-induced increase in intestinal epithelial tight junction permeability via a TLR-2 and PI3K-dependent inhibition of NF-κB activation. Front. Immunol..

[150] Zhou J. (2022). Programmable Probiotics Modulate Inflammation and Gut Microbiota for Inflammatory Bowel Disease Treatment after Effective Oral Delivery. Nat. Commun..

[151] Swanson K.S., Gibson G.R., Hutkins R., Reimer R.A., Reid G., Verbeke K., Scott K.P., Holscher H.D., Azad M.B., Delzenne N.M. (2020). The International Scientific Association for Probiotics and Prebiotics (ISAPP) Consensus Statement on the Definition and Scope of Synbiotics. Nat. Rev. Gastroenterol. Hepatol..

[152] Paul P. (2022). The Effect of Microbiome-Modulating Probiotics, Prebiotics and Synbiotics on Glucose Homeostasis in Type 2 Diabetes: A Systematic Review, Meta-Analysis, and Meta-Regression of Clinical Trials. Pharmacol. Res..

[153] Ji J., Jin W., Liu S., Jiao Z., Li X. (2023). Probiotics, Prebiotics, and Postbiotics in Health and Disease. MedComm.

[154] Wang Y.-T., Yang P.-C., Zhang Y.-F., Sun J.-F. (2024). Synthesis and Clinical Application of New Drugs Approved by FDA in 2023. Eur. J. Med. Chem..

[155] Carson M.D., Warner A.J., Geiser V.L., Hathaway-Schrader J.D., Alekseyenko A.V., Marshall J., Westwater C., Novince C.M. (2023). Prolonged Antibiotic Exposure during Adolescence Dysregulates Liver Metabolism and Promotes Adiposity in Mice. Am. J. Pathol..

[156] Wang Y., Tang J., Lv Q., Tan Y., Dong X., Liu H., Zhao N., He Z., Kou Y., Tan Y. (2022). Establishment and Resilience of Transplanted Gut Microbiota in Aged Mice. iScience.

[157] Schenck L.P., Beck P.L., MacDonald J.A. (2015). Gastrointestinal dysbiosis and the use of fecal microbial transplantation in Clostridium difficile infection. World J. Gastrointest Pathophysiol..

[158] Yu E.W., Gao L., Stastka P., Cheney M.C., Mahabamunuge J., Soto M.T., Ford C.B., Bryant J.A., Henn M.R., Hohmann E.L. (2020). Fecal Microbiota Transplantation for the Improvement of Metabolism in Obesity: The FMT-TRIM Double-Blind Placebo-Controlled Pilot Trial. PLoS Med..

[159] Rasmussen T.S., Mentzel C.M.J., Kot W., Castro-Mejía J.L., Zuffa S., Swann J.R., Hansen L.H., Vogensen F.K., Hansen A.K., Nielsen D.S. (2020). Faecal Virome Transplantation Decreases Symptoms of Type 2 Diabetes and Obesity in a Murine Model. Gut.

[160] Wu G., Xu T., Zhao N., Lam Y.Y., Ding X., Wei D., Fan J., Shi Y., Li X., Li M. (2024). A core microbiome signature as an indicator of health. Cell.

